# LMP2 and TAP2 impair tumor growth and metastasis by inhibiting Wnt/β-catenin signaling pathway and EMT in cervical cancer

**DOI:** 10.1186/s12885-023-11639-y

**Published:** 2023-11-20

**Authors:** Zhengyan Cheng, Hongbo Wang, Zewei Yang, Jiaxu Li, Xing Chen

**Affiliations:** 1 Department of Pathology, Eastern Hospital, Sichuan Academy of Medical Science & Sichuan Provincial People’s Hospital, Chengdu, 610101 China; 2 Department of Thoracic Surgery, Eastern Hospital, Sichuan Academy of Medical Science & Sichuan Provincial People’s Hospital, Chengdu, 610101 China

**Keywords:** Cervical carcinoma, LMP2, TAP2, Oncogenesis, EMT, Wnt1

## Abstract

**Background:**

The roles of low molecular mass polypeptide 2 (LMP2) and transporter-associated with antigen processing (TAP2) in tumorigenesis are controversial. Here we aimed to explore the effect of LMP2 and TAP2 on the oncogenesis and metastasis of cervical cancer cells.

**Methods:**

The expressions of LMP2 and TAP2 in cervical cancer and normal tissues were determined by qPCR. Plate colony formation, cell counting kit-8 analysis and in vivo tumor xenograft assays were used to detect the tumor growth. Wound healing and transwell assays were used to detect the metastasis of cervical cancer. Gelatin zymography and western blotting assays were used to detect the effect of LMP2 and TAP2 on the EMT and Wnt/β-catenin pathway in cervical cancer cells.

**Results:**

In the present study, we reported that LMP2 and TAP2 levels were overexpressed in cervical cancer. Overexpression of LMP2 and TAP2 impaired the proliferation of Hela cells. In vivo studies substantiated that LMP2 and TAP2 antagonized tumor growth. Likewise, LMP2 and TAP2 overexpression decreased the migration and invasion ability of Hela cells by regulating the process of epithelial-mesenchymal transition (EMT). Mechanically, LMP2 and TAP2 subverted the protein abundance of Wnt1 and β-catenin, thereby downregulating their downstream targets Cyclin D1 and c-Myc. In addition, Wnt1 overexpression partially rescued the observed consequences of ectopic expression of LMP2 and TAP2 in cervical cancer cells. Taken together, our study revealed that LMP2 and TAP2 suppress the oncogenesis and metastasis of cervical cancer cells by Wnt/β-catenin pathway and altering EMT.

**Conclusion:**

LMP2 and TAP2 may inhibit the oncogenesis and metastasis of cervical cancer cells by inhibiting the process of EMT and the Wnt/β-catenin signaling pathway, which may provide important insight into prospective targets for the treatment of cervical cancer.

**Supplementary Information:**

The online version contains supplementary material available at 10.1186/s12885-023-11639-y.

## Background

Cervical cancer (CC), mostly induced by human papillomavirus (HPV), ranks fourth most common cancer in women and affects women aged between 30 and 39 years and 60 and 69 years of age for both incidence and mortality. Further, it is frequently diagnosed among women between ages 30 and 39 years [1, 2]. Since there are no typical symptoms and signs in the early stage of cervical cancer, it is easy to be misdiagnosed or missed, which seriously threatens the life and health of women [3]. Despite the remarkable recent advances in the prevention, screening and diagnosis of cervical cancer, which have allowed early detection and treatment, leading to a marked decrease in the morbidity and mortality of cervical cancer, the 5-year survival rate after treatment of patients with advanced cervical cancer is still unsatisfactory [4]. Therefore, it is urgent to develop a treatment that can significantly inhibit the progression of cervical cancer and improve the survival of patients with cervical cancer.

The proliferation and metastasis of cervical cancer are closely related to the abnormal activation of epithelial-mesenchymal transition (EMT) [5]. Phenotypic changes from epithelial cells to mesenchymal cells play a decisive role in the process of metastasis which involves the upregulation of α-smooth muscle actin (α-SMA) and vimentin and downregulation of E-cadherin induced by EMT [6–8]. A number of studies have shown that the occurrence of EMT in cervical cancer increases the subpopulation of cancer stem cells (CSC) with the high metastatic potential of cervical cancer [9, 10]. Hence, inhibiting EMT of cervical cancer cells can increase their sensitivity to radiation and drugs, and this sensitization can improve the survival rate of cervical cancer patients [11, 12].

In recent years, it has been found that the classical Wnt/β-catenin signaling pathway plays an essential role in the regulation of various physiological processes, including stem cell self-renewal, early embryonic development, organogenesis, tissue regeneration, and is closely related to the occurrence and development of multiple human tumor diseases [13, 14]. The mutation of *Wnt* genes or the alteration of Wnt-related ligand expression leads to the occurrence and development of cancer, such as hepatocellular carcinoma (HCC), breast cancer, melanoma and colorectal cancer [15–17]. Additionally, Cyclin D1 and c-Myc have been revealed to be strictly regulated by the Wnt/β-catenin signaling pathway [18].

The major histocompatibility complex (MHC) class I antigen-presenting pathway is strictly correlated with peptide delivery (and subsequent binding to MHC-I peptides) to promote the clearance process of tumor cells. LMP/TAP system can recognize tumor antigens and play an important role in host anti-tumor immune protection through MHC-I molecules and cytotoxic T lymphocytes (CTL) [19, 20]. Nevertheless, the specific role and underlying mechanism of LMP2 and TAP2 in the oncogenesis and metastasis of cervical cancer have not been elucidated.

In this study, the mechanism of LMP2 and TAP2 impairing the oncogenesis and metastasis of cervical cancer was investigated in vitro and in vivo, which lays the foundation for the research of cervical cancer gene vaccine and dual gene nucleic acid vaccine.

## Methods

### Cell lines

HeLa (ATCC CCL-2) cell lines and HEK293T were purchased from the American Type Culture Collection (Manassas, VA) and were grown in Dulbecco’s modified Eagle’s medium (DMEM; Life Technologies) supplemented with 10% FBS.

### Reagents

The antibodies used in this study were as follows: anti-LMP2 (PA1-1960, Invitrogen, WB, 1:2000), anti-TAP2 (PA5-37414, Invitrogen, WB, 1:1000), anti-MMP2 (10373-2-AP, Proteintech, WB, 1:500), anti-MMP9 (27306-1-AP, Proteintech, WB, 1:1000), anti-Vimentin (10366-1-AP, Proteintech, WB, 1:2000), anti-E-cadherin (20874-1-AP, Proteintech, WB, 1:5000), anti-N-cadherin (22018-1-AP, Proteintech, WB, 1:2000), anti-Cyclin D1 (26939-1-AP, Proteintech, WB), anti-c-Myc (67447-1-Ig, Proteintech, WB, 1:2000), anti-Wnt1 (ab15251, Abcam, WB, 1:1000), anti-Wnt4 (ab262696, Abcam, WB, 1:1000), anti-β-catenin (8480, Cell Signaling Technology, WB, 1:1000), anti-GAPDH (60004-1-Ig, Proteintech, WB, 1:50000) and anti-β-tubulin (10094-1-AP, Proteintech, WB, 1:1000).

### LMP2 and TAP2 overexpression (OE) and knockdown (KD)

The *LMP2* and *TAP2* genes were PCR amplified from Hela cells cDNA using specific primers with an XhoI restriction enzyme site at the 5’-end and a NotI site at the 3’-end. The fragments were cloned into the vector pcDNA3.1. Primers are as follows: *Lmp2*, F: ATGCTGCGGGCGGGAGCA, R: TCACTCATCATAGAATTTTGGCAGTTC and *Tap2*, F: ATGCGGCTCCCTGACCTG, R: TCAGTCCATCAGCCGCTG.

A total of 2 × 10^5^ cells were seeded into each well of a 6-well plate and grown for 12 h, followed by transfection with the indicated plasmids, which were delivered using Lipofectamine 3000 transfection reagents (Thermo Fisher, Waltham, Massachusetts, USA) according to the manufacturer’s instructions.

Lentiviral particles for shLMP2 and shTAP2 were produced by transfection of 293T cells with pLKO (shRNA), psPAX2 (packaging), and pMD2.G (envelope) plasmids (1:0.65:0.35 ratio) using jetPRIME according to manufacturer’s instruction. The supernatant was filtered and added to Hela cells mixed with 8 µg/ml Polybrene overnight. Then, the supernatant was replaced with DMEM/10% FBS. shRNA sequences are listed as followed: *LMP2* (shLMP2: 5’- CATCGAGTCATCTTGGGCAAT-3’) and *TAP2* (shTAP2: 5’-CGGTTCTGTGAGGAACAACAT-3’).

### RNAi interference

In the present study, the siRNA sequences designed to target Wnt1 were as follows: Wnt1 siRNA#1, 5’-CTGCAGCTGTTGAGCCGCAAA-3’, and Wnt1 siRNA#2, 5’-CTCGCGCGTCCTGTACGGCAA-3’. As per the manufacturer’s instructions, Lipofectamine RNAiMAX reagent (Invitrogen) was used for transfecting HeLa cells with indicated siRNAs (40 nM). Following transfection for 24 h, subsequent experiments were conducted.

### Cell counting kit-8 (CCK-8) assay

The cell viability was detected by CCK-8 assay (Beyotime) according to the manufacturer’s instructions. In brief, Hela cells were seeded into 96-well plates at a density of 5 × 10^3^ cells/well and transfected for 24 and 48 h, followed by incubation with 10 uL of CCK-8 solution at 37 °C for 2 h. The absorbance was determined at a wavelength of 450 nm.

### Western blotting assay

The transfected cells were harvested and lysed with NP-40 supplemented with a protease inhibitor cocktail. Equal amounts of proteins were separated by SDS-PAGE and then transferred to a polyvinylidene difluoride (PVDF) membrane, followed by blocking with 5% nonfat milk in PBS/T with 0.1% polysorbate-20. After washing three times with PBS/T, the membranes were probed with the indicated primary antibodies at 37 °C for 4 h and horseradish peroxidase (HRP)-conjugated secondary antibodies (Beyotime, China) for 45 min at room temperature. Proteins were visualized by enhanced chemiluminescence (ECL) reagents (Beyotime).

### Plate colony formation analysis

After cell transfection, cells in each group at the logarithmic growth stage were digested with trypsin and beaten into single cells. The cells were suspended in a DMEM medium containing 10% fetal bovine serum (FBS) for standby and counting. The cell suspension was diluted in gradient multiple dilutions, and 1000 cells/well were inoculated into a 2 ml medium. The cells were placed in a 37℃ 5% CO_2_ cell culture box with saturated humidity for 1 week. It is often observed that when visible clones appear in the petri dish, the culture is terminated and the number of clones larger than 10 cells is counted under the microscope. Finally, the clone formation rate was calculated by Image J.

### Migration assay

For the scratch wound assay, the cells of the control group, LMP2 group, TAP2 group and LMP2 + TAP2 group were seeded into 12-well plates with 1 × 10^5^ cells in each well for 24 h. Then 200 µL pipette tip was used to draw straight lines, and the scratched cells were washed three times with PBS, followed by the culture with serum-free medium. The scratch width was measured 24 and 48 h later, and the scratch width ratio before and after the experiment was calculated.

For the transwell assay, a total of 1 × 10^5^ serum-free cells were added into each transwell chamber with matrigel, and 2 parallel multiple pores were set. 500 µL 10% FBS DMEM was added into the lower chamber, and the cells were stained with 1% crystal violet, and the number of migrating and invading cells in the field of vision was observed under a 100× microscope.

### In vivo tumor xenograft assay

In brief, a total of twenty female Balb/c mice (5-week-old, 20 g, purchased from Chengdu Dashuo Laboratory Animal) were randomly grouped into four groups: Scramble group (n = 5), LMP2 group (n = 5), TAP2 group (n = 5) and L + T group (n = 5). Mice were separately subcutaneously inoculated with 2 × 10^6^ LMP2, TAP2 or L + T knockdown Hela cells. After inoculation, nude mice were kept at 24 ± 2˚C for 28 days in a 12 h light/dark cycle room with *ad libitum* access to food and water. At the end of the trial, nude mice were sacrificed by anesthesia using 3–5% isoflurane. Tumor sizes were measured using calipers and tumor volumes were calculated by the formula as follows: V = (L × W^2^)/2 [21].

### Statistical analysis

Statistical differences were determined by one-way analyses of variance with Tukey’s multiple comparison test using GraphPad Prism 8.0 software. For all experiments, differences were considered statistically significant when *P* values were < 0.05.

## Results

### LMP2 and TAP2 levels are upregulated in cervical cancer tissues

**Fig. 1:**
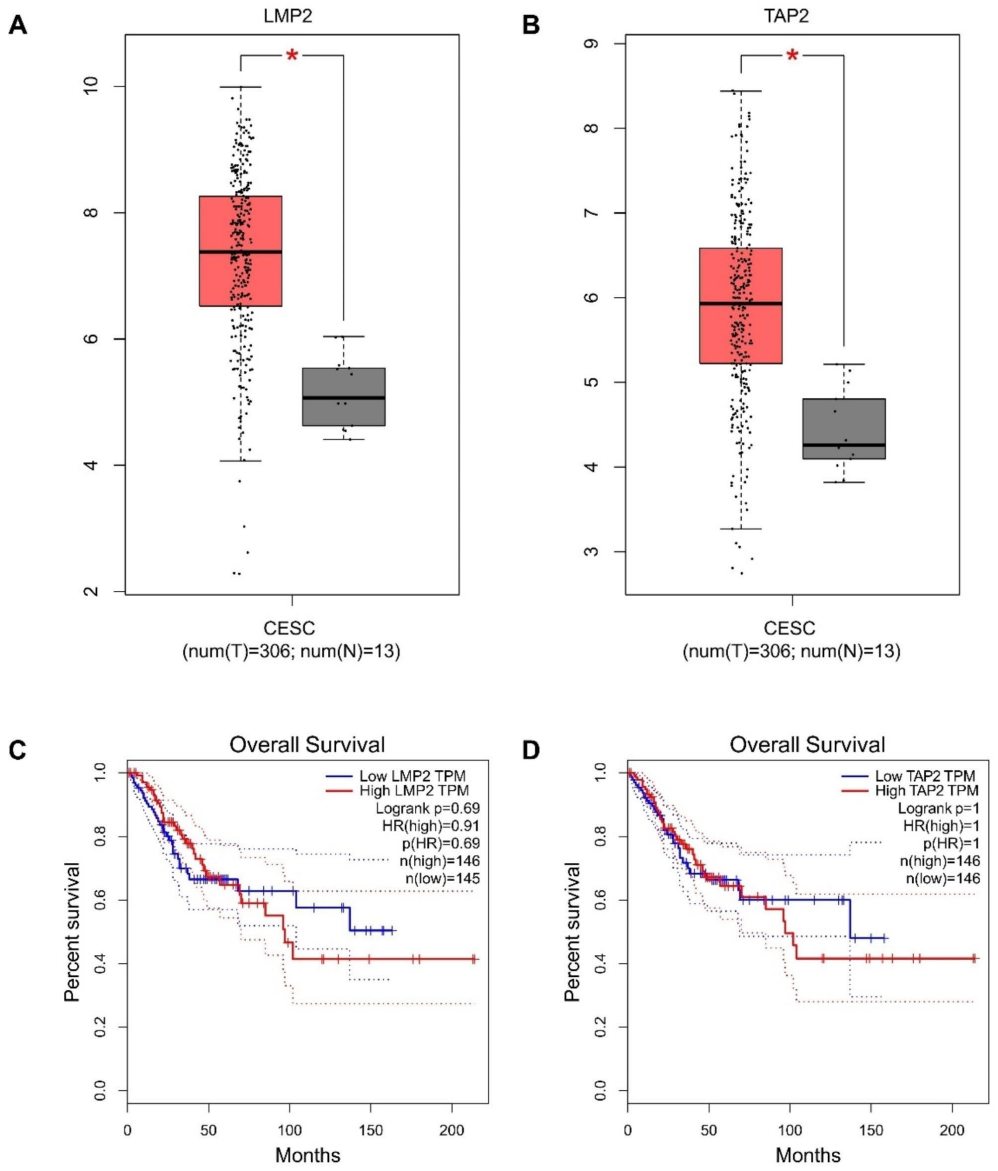
The expressions of LMP2 and TAP2 in cervical cancer. **A** The level of LMP2 mRNA in cervical cancer tissues compared with normal tissues is shown in the TCGA database. **B** The level of TAP2 mRNA in cervical cancer tissues compared with normal tissues is shown in the TCGA database. **C** Percent of overall survival with low LMP2 expression was higher than that with high LMP2 expression (Data source: GEPIA: http://gepia.cancer-pku.cn/). **D** Percent of overall survival with low TAP2 expression was higher than that with high TAP2 expression (Data source: GEPIA: http://gepia.cancer-pku.cn/)

To investigate the clinical significance of LMP2 and TAP2 in cervical cancer, we conducted an investigation using the online Gene Expression Profiling Interactive Analysis (GEPIA) tool (http://gepia.cancer-pku.cn/) on The Cancer Genome Atlas (TCGA) database. Our analysis compared the transcriptomic profiles of cervical cancer and normal cervical tissues. The results revealed a significant upregulation of LMP2 and TAP2 mRNA levels in cervical carcinoma tissue compared to normal tissue (Fig. 1A and B). Furthermore, Kaplan-Meier survival analysis demonstrated that patients with high expression levels of LMP2 and TAP2 had a poorer overall survival in comparison to patients with low expression levels (Fig. 1C and D).

### LMP2 and TAP2 negatively regulate tumor growth in vitro and in vivo

**Fig. 2:**
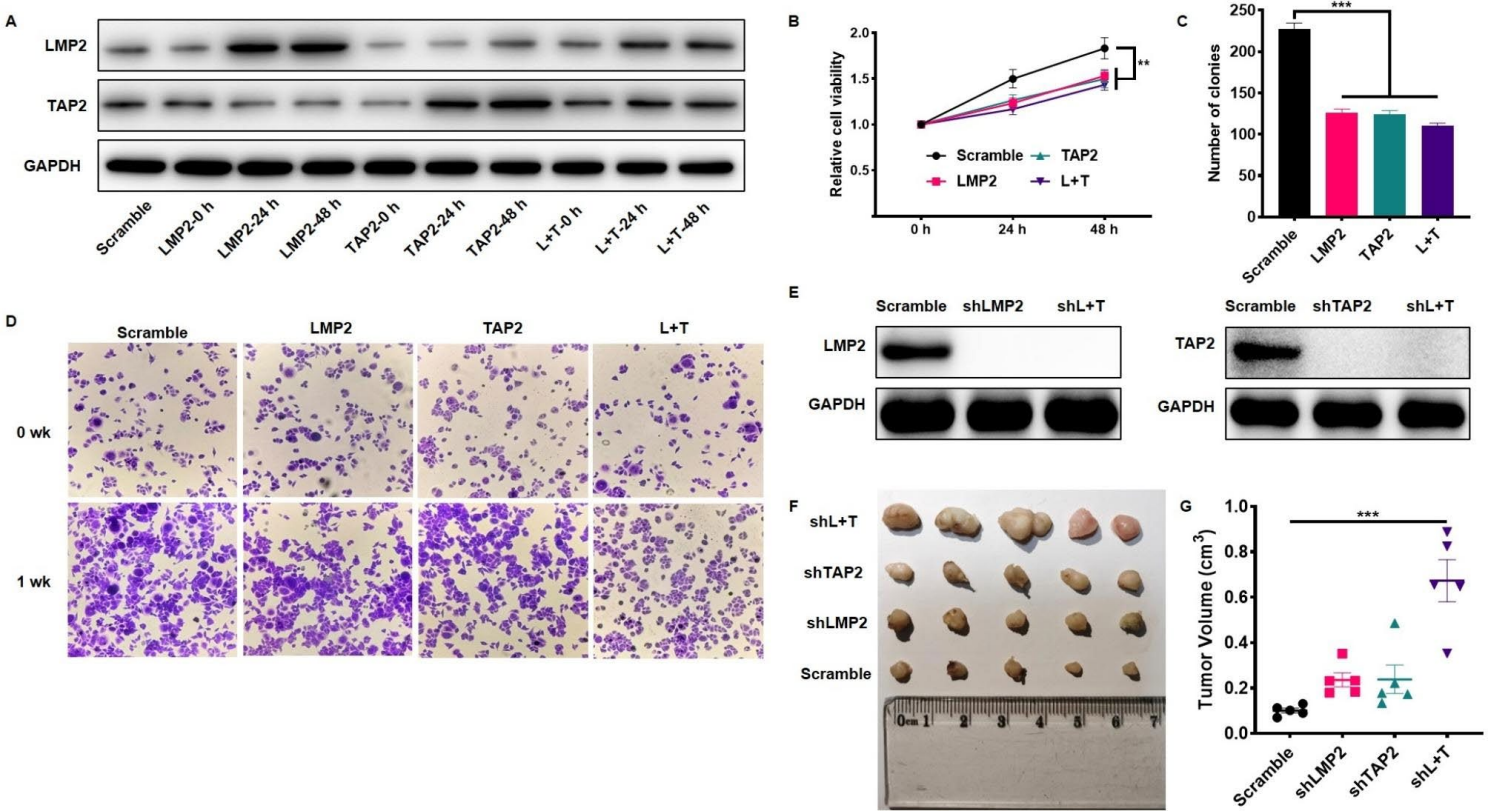
LMP2 and TAP2 inhibit the tumor growth of cervical cancer *in vitro and in vivo*. **A** Western blot analysis for the protein abundances of LMP2 and TAP2 after LMP2 and TAP2 plasmids transfection for 24 and 48 h. **B** The relative cell viability of Hela cells using the CCK-8 assay. **C** The statistical analysis of the number of colonies. **D** The plate colony formation assay for the proliferation of Hela cells transfected with LMP2 and TAP2 plasmids. **E** Western blot analysis for the validation of knockdown of LMP2 and TAP2. **F** The knockdown of LMP2 and TAP2 promotes tumor growth in vivo. **G** The statistical analysis of the tumor volume. (**) *P* < 0.01, and (***) *P* < 0.001 compared with the scramble group

To examine the effect of LMP2 and TAP2 on the oncogenesis of cervical cancer cells, the pcDNA3.1-LMP2 and pcDNA3.1-TAP2 plasmids were constructed and transfected into Hela cells, and the ectopic expression of LMP2 and TAP2 was validated by qRT-PCR (Supplementary Fig. 1) and western blotting (Fig. 2A). A significant decrease in cell viability (*P* < 0.01) was caused by LMP2 and TAP2 overexpression (Fig. 2B). Plate colony experiments (Fig. 2C and D) also demonstrated that LMP2 and TAP2 overexpression decreased the number of Hela cell colony formation (*P* < 0.01).

Since LMP2 and TAP2 impair tumor growth and proliferation in vitro, we subsequently detected the effects of LMP2 and TAP2 on the oncogenesis of cervical cancer cells in vivo. Stable LMP2 and TAP2-knockdown Hela cells using LMP2 and TAP2 shRNAs were constructed (Fig. 2E and supplementary Fig. 2) and subcutaneously inoculated into nude mice for 28 days. As shown in Fig. 1F and G, the shRNA-mediated knockdown of LMP2 and TAP2 markedly enhanced the tumor size compared to the scramble group (*P* < 0.01).

### LMP2 and TAP2 negatively regulate the metastasis of cervical cancer cells

**Fig. 3:**
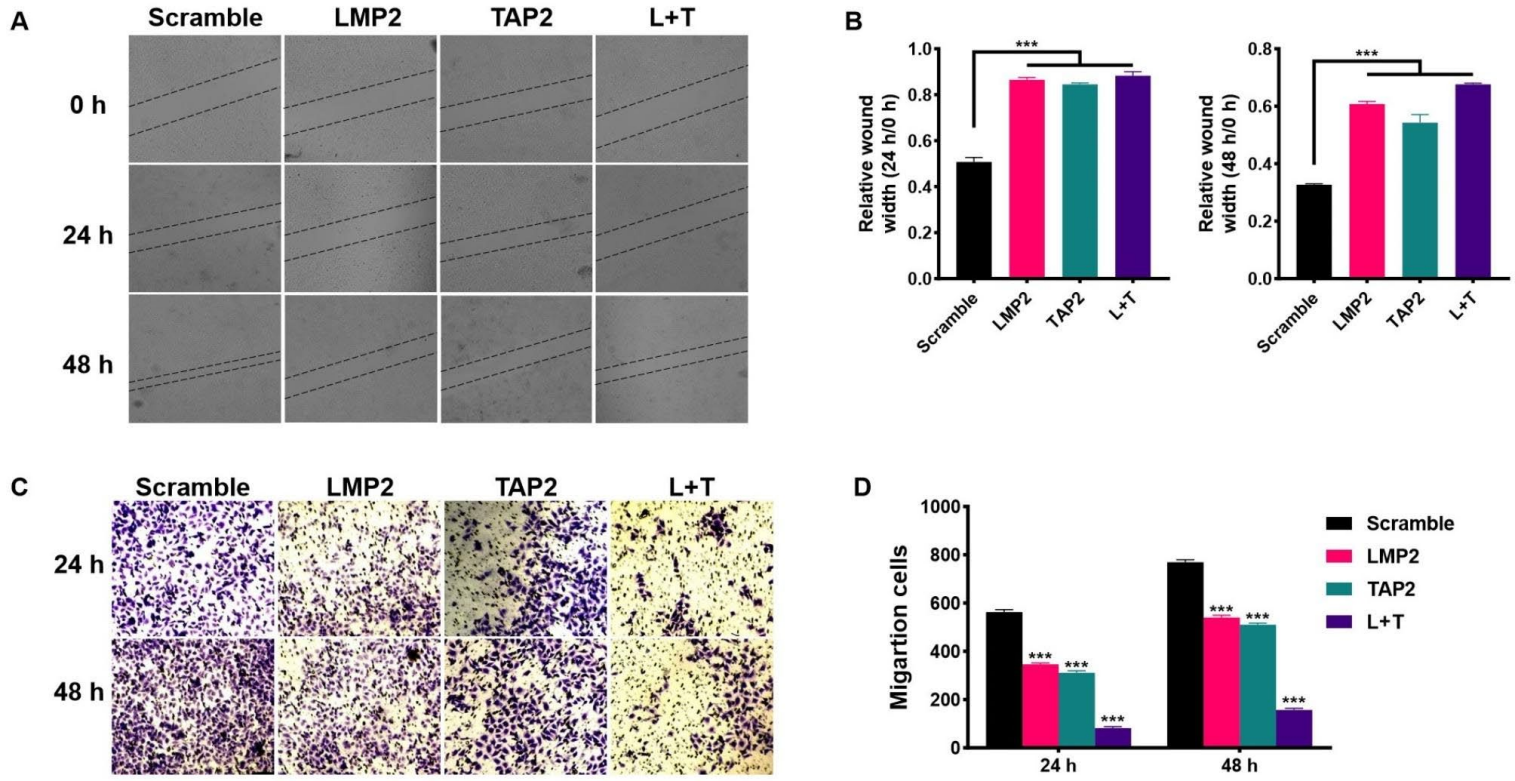
The effect of LMP2 and TAP2 overexpression on the migration and invasion of cervical cancer cells. **A** The wound-healing assay for the migration ability of Hela cells transfected with LMP2 and TAP2 plasmids for 24 and 48 h. **B** The statistical analysis of relative wound width of Hela cells. **C** The transwell assay for the invasion ability of Hela cells transfected with LMP2 and TAP2 plasmids for 24 and 48 h. **D** The statistical analysis of migration cells. (***) *P* < 0.001 compared with the scramble group

The effect of LMP2 and TAP2 on the migration ability of cervical cancer cells was detected by cell scratch test. The cell scratch width was measured 24 and 48 h later and the scratch width ratio before and after the experiment was calculated. The results showed that the wound width of Hela cells in the control group was significantly lower (*P* < 0.01) than that of cervical cancer cells after LMP2 and TAP2 transfection (Fig. 3A and B). These experiments suggested that LMP2 and TAP2 could inhibit the migration of cervical cancer cells.

To further confirm the above results, the transwell experiment was used to explore the effect of LMP2 and TAP2 on the invasion ability of cervical cancer cells. The results showed that compared with the control group, the invasion of Hela cells was significantly decreased (*P* < 0.01) after LMP2 and TAP2 transfection (Fig. 3C and D).

### LMP2 and TAP2 regulate EMT in cervical cancer cells

**Fig. 4:**
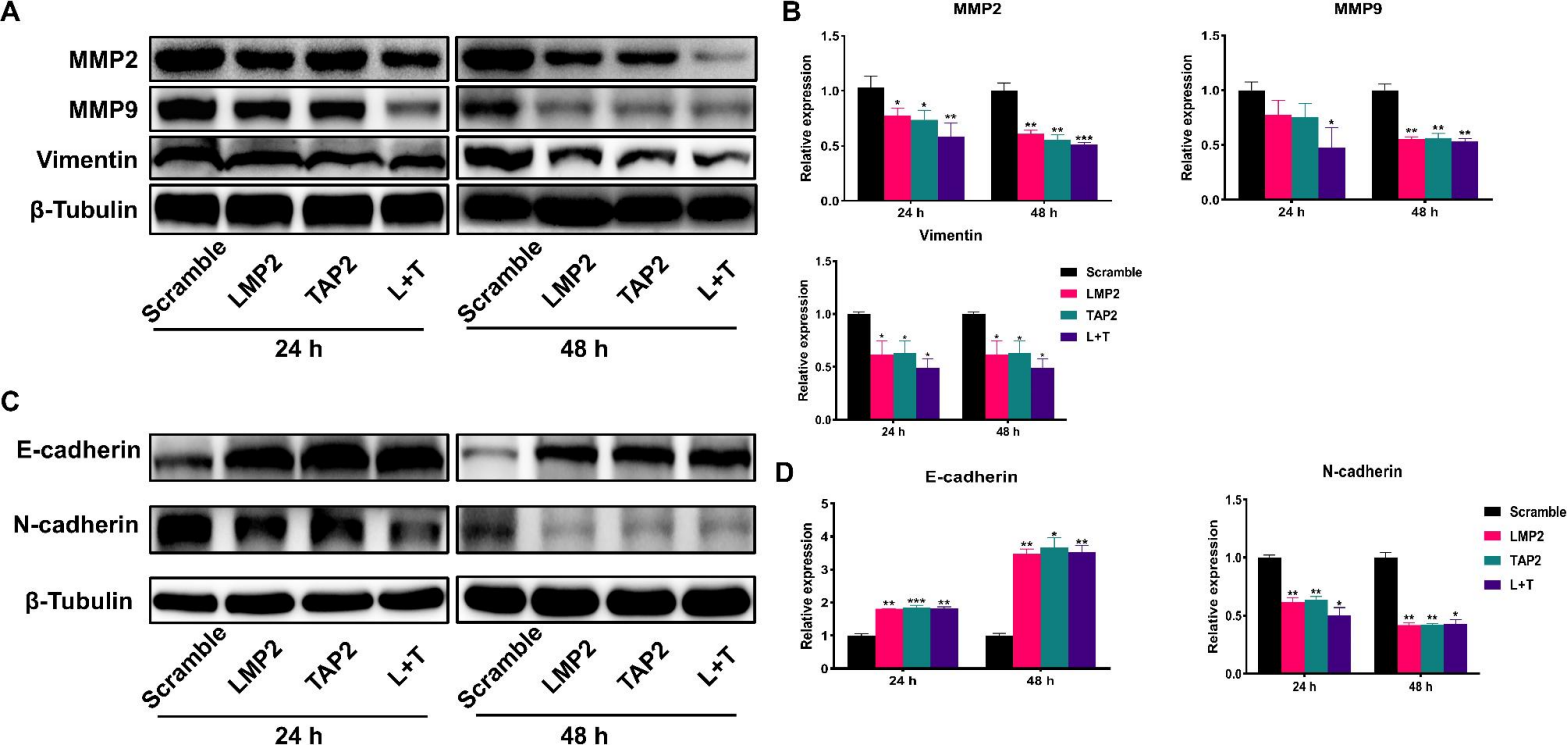
LMP2 and TAP2 negatively regulate EMT in cervical cancer cells. **A** Western blot analysis for the protein abundances of MMP2, MMP9 and Vimentin in Hela cells transfected with LMP2 and TAP2 plasmids for 24 and 48 h. **B** The statistical analysis of the fold change of MMP2, MMP9 and Vimentin. **C** Western blot analysis for the protein abundances of E-cadherin and N-cadherin in Hela cells transfected with LMP2 and TAP2 plasmids for 24 and 48 h. **D** The statistical analysis of the fold change of E-cadherin and N-cadherin. (*) *P* < 0.05, (**) *P* < 0.01 and (***) *P* < 0.001 compared with the scramble group

EMT is widely involved in various tumor metastasis processes. In this study, LMP2 and TAP2 regulated the expression of EMT-related proteins. The results showed that LMP2 and TAP2 overexpression significantly downregulated (*P* < 0.01) the protein abundances of MMP2/9 and vimentin in Hela cells (Fig. 4A and B), which was confirmed by gelatin zymography assay (Supplementary Fig. 3). In addition, LMP2 and TAP2 overexpression significantly promoted the protein abundances of E-cadherin and inhibited (*P* < 0.01) the expression of N-cadherin in Hela cells (Fig. 4C and D). In conclusion, LMP2 and TAP2 may regulate the migration of cervical cancer cells by participating in the regulation of EMT.

### LMP2 and TAP2 inhibit Wnt/β-catenin pathway in cervical cancer

**Fig. 5:**
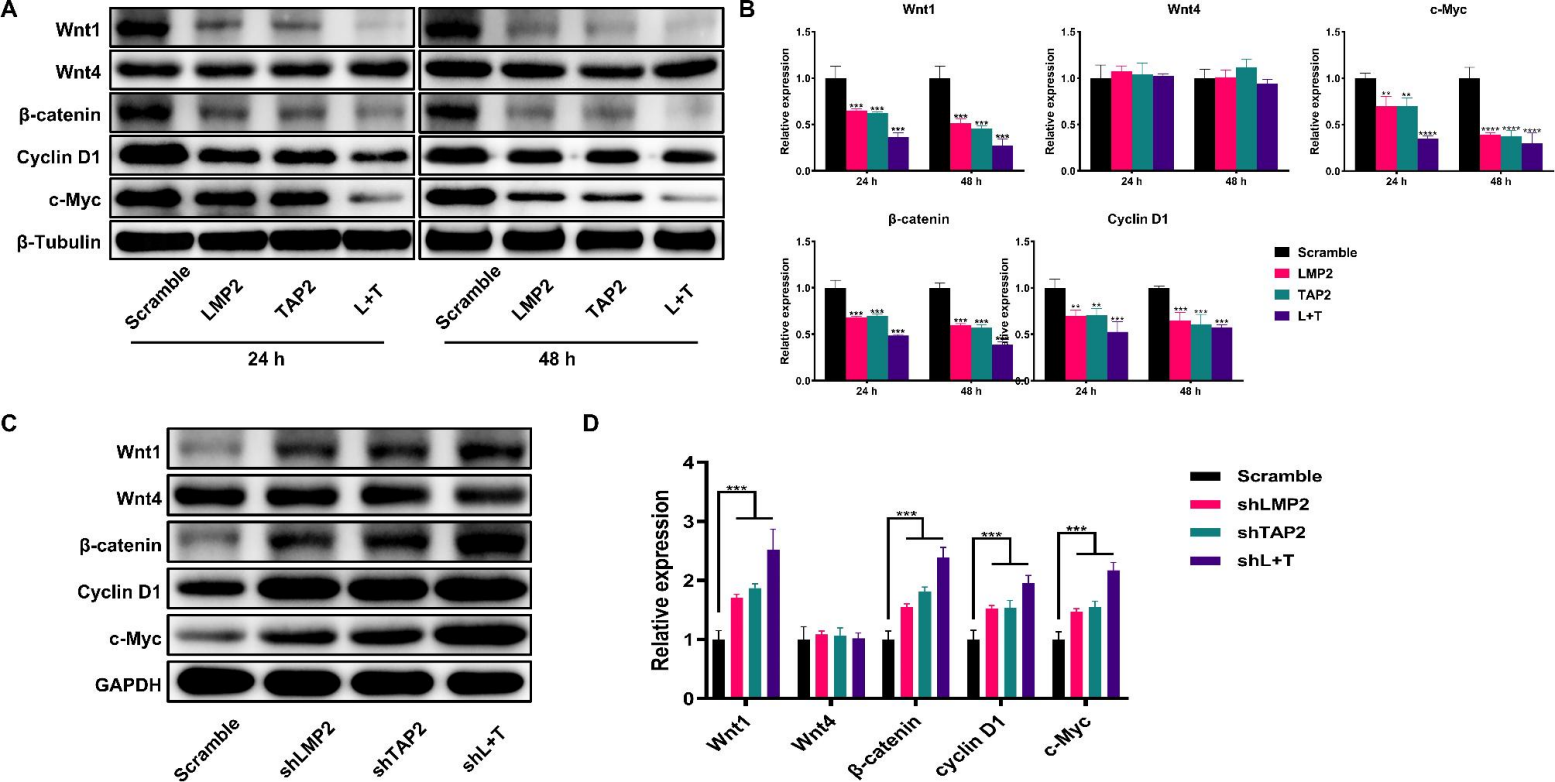
LMP2 and TAP2 attenuate the Wnt/β-catenin signaling pathway in cervical cancer cells. **A** Western blot analysis for the protein abundances of Wnt1, Wnt4, β-catenin, Cyclin D1 and c-Myc in Hela cells transfected with LMP2 and TAP2 plasmids for 24 and 48 h. **B** The statistical analysis of the fold change of Wnt1, Wnt4, β-catenin, Cyclin D1 and c-Myc. **C** Western blot analysis for the protein abundances of Wnt1, Wnt4, β-catenin, Cyclin D1 and c-Myc in LMP2 and TAP2-KD Hela cells. **D** The statistical analysis of the fold change of Wnt1, Wnt4, β-catenin, Cyclin D1 and c-Myc. (**) *P* < 0.01, (***) *P* < 0.001 and (****) *P* < 0.0001 compared with the scramble group

To gain insight into the underlying mechanism of LMP2 and TAP2 regulating tumorigenicity and EMT, we focused on the Wnt/β-catenin pathway since its role in the process of oncogenesis. Notably, our results showed that LMP2 and TAP2 suppressed (*P* < 0.01) the protein levels of Wnt1 and β-catenin but not Wnt4 (Fig. 5A and B). Conversely, the knockdown of LMP2 and TAP2 dramatically promoted (*P* < 0.01) the protein levels of Wnt1 and β-catenin but not Wnt4 (Fig. 5C and D), further suggesting that LMP2 and TAP2 play a crucial role in regulating the Wnt/β-catenin pathway. In addition, downstream target genes, including Cyclin D1 and c-Myc were altered by LMP2 and TAP2, with a stronger influence by LMP2 and TAP2 co-transfection or co-knockdown (*P* < 0.01). The results were also substantiated in the in vivo study (Supplementary Fig. 4). Our findings suggested that LMP2 and TAP2 possibly modulate the Wnt/β-catenin pathway to impair the proliferation of cervical cancer cells.

### The decreased proliferation of cervical cancer cells caused by LMP2 and TAP2 is mainly dependent on Wnt1

**Fig. 6:**
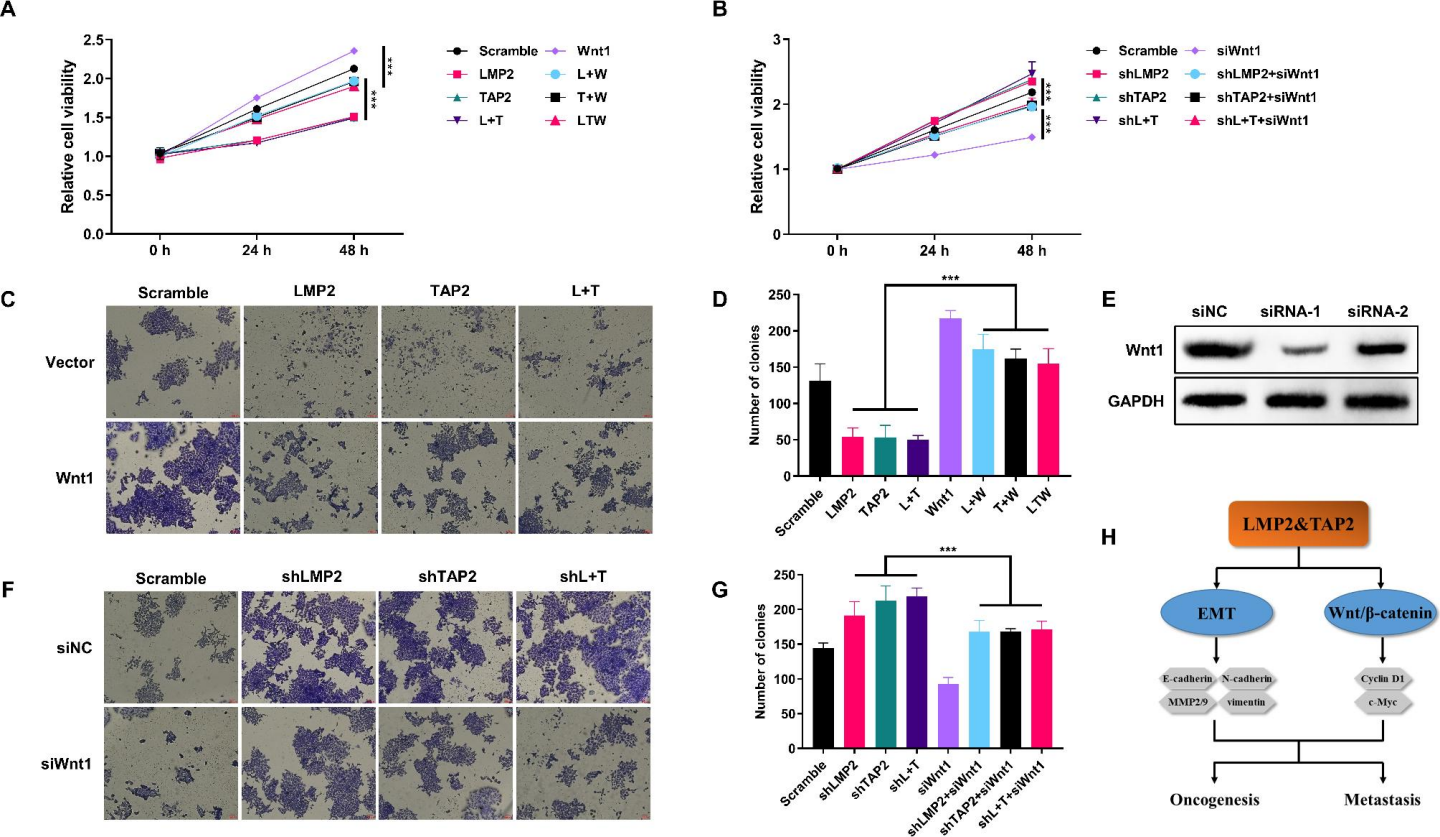
The decreased proliferation of cervical cancer cells caused by LMP2 and TAP2 is mainly dependent on Wnt1. **A** The relative cell viability of Hela cells transfected with LMP2, TAP2 and Wnt1 plasmids for 24 and 48 h using the CCK-8 assay. **B** The relative cell viability of LMP2, TAP2-depleted Hela cells transfected with siRNA of Wnt1 for 24 and 48 h using the CCK-8 assay. **C** The plate colony formation assay for the proliferation of Hela cells transfected with LMP2, TAP2 and Wnt1 plasmids. **D** The statistical analysis of the number of colonies. **E** Western blot analysis for the protein abundances of Wnt1 in Hela cells transfected with siRNA for Wnt1. **F** The plate colony formation assay for the proliferation of LMP2, TAP2-depleted Hela cells transfected with siRNA for Wnt1. **G** The statistical analysis of thenumber of colonies. (***) *P* < 0.001 compared with the scramble group. **H** The schematic diagram of the mechanism of LMP2 and TAP2 impairs tumor growth and metastasis by inhibiting the Wnt/β-catenin signaling pathway and EMT in cervical cancer

To explore whether the presence of Wnt1 is indeed necessary for the observed effects resulting from the overexpression of LMP2 and TAP2 in cervical cancer cells, we introduced exogenous expression of Wnt1 into LMP2 and/or TAP2 overexpressed cells. The results depicted in Fig. 6A, C and D demonstrate that the impairments in cell growth and proliferation induced by LMP2 and TAP2 were partially rectified by overexpressing Wnt1. Moreover, we also transfected Wnt1 siRNAs into cells that were depleted of LMP2 and/or TAP2 (Fig. 6E). The increased tumor growth resulting from LMP2 and/or TAP2 depletion was also partially reversed by suppressing Wnt1 levels (Fig. 6B, F and G). Consequently, these findings indicate that the primary mechanisms through which LMP2 and TAP2 exert their biological effects in cervical cancer predominantly involve the regulation of Wnt1.

## Discussion

Cervical cancer poses a serious threat to women’s health and life, arising mostly (more than 95%) from persistent infection of high-risk HPV, which can be detected in 99.8% of cervical cancer specimens [22, 23]. Therefore, based on the current situation of cervical cancer, we propose to develop a therapeutic target that can alter the host immune response.

Studies have found that LMP and TAP gene polymorphisms are associated with the occurrence and development of malignant tumors and autoimmune diseases due to their roles in the processing and presentation of endogenous antigens [24–28]. LMP/TAP system can recognize tumor antigens and play an important role in host anti-tumor immune protection through MHC-I molecules and CTL [29]. At present, there are many studies on LMP or TAP for nasopharyngeal cancer, gliomas, lymphoma and autoimmune diseases [30–32]. However, the effect of the LMP/TAP combination on the cervical cells is poorly understood.

In the present study, we found that the proliferation and migration ability of cervical cancer were significantly reduced in the LMP2 and TAP2 group compared with the control group, especially in LMP2 and TAP2 co-transfection group. As an important physiological mechanism, EMT not only affects the growth and development of embryos but also plays an important role in chronic inflammation and tumor metastasis [33]. Studies have demonstrated that knockdown of FTS (Fused Toes Homolog), an oncogene involved in cervical cancer pathogenesis, suppresses cell migration by downregulating MMP-2 and MMP-9 in cervical cancer [34]. Based on the above results, we observed that the levels of MMP2, MMP9, vimentin, N-cadherin and E-cadherin were remarkably altered in LMP2 and TAP2 transfected cervical cancer cells, suggesting that LMP2 and TAP2 restrict the process of EMT in cervical cancer cells.

Recent studies have found that abnormal activation of the Wnt/β⁃catenin pathway is associated with the migration and invasion of various tumors, including skin cancer, colorectal cancer, breast cancer and cervical cancer [14, 35, 36]. It is reported that the knockdown of NEK2 can reduce the expression of Wnt1 and β-catenin to subvert the oncogenesis of cervical cancer cells [37]. In this study, we found that the knockdown of LMP2 and TAP2 promoted the expression of Wnt1 and β-catenin and their downstream targets c-Myc and Cyclin D1 at translation level in Hela cells. Additionally, our findings strongly suggest that LMP2 and TAP2’s effects on cell growth, proliferation, and tumor growth in cervical cancer are closely associated with the modulation of Wnt1 expression. The introduction of exogenous Wnt1 partially counteracted the negative impacts of LMP2 and TAP2 overexpression, while the inhibition of endogenous Wnt1 attenuated the tumor-promoting effects of LMP2 and TAP2 depletion, highlighting the pivotal role of the Wnt1 pathway in mediating the biological functions of LMP2 and TAP2 in cervical cancer cells. However, the LMP2 and TAP2 were not rescued in the LMP2 and TAP2-knockdown cells and it is still required for further study in the in vivo study by overexpression methods. Nevertheless, further research is needed to elucidate the exact molecular crosstalk between LMP2, TAP2, and the Wnt1 pathway to provide a comprehensive understanding of their intricate interactions and potential clinical implications. Importantly, we proved that LMP2 and TAP2 overexpression attenuated the Wnt/β-catenin signaling pathway.

## Conclusions

As proposed in Fig. 6H, LMP2 and TAP2 may inhibit the oncogenesis and metastasis of cervical cancer cells by impairing the process of EMT and the Wnt/β-catenin signaling pathway, which will provide new targets and therapeutic strategies for the treatment of cervical cancer.

### Electronic supplementary material

Below is the link to the electronic supplementary material.

**Supplemental figures: Supplementary Fig. 1** The relative mRNA levels of Lmp2 and Tap2 were determined by RT-qPCR in LMP2 and TAP2 overexpression (OE) cells. **Supplementary Fig. 2** The relative mRNA levels of Lmp2 and Tap2 were determined by RT-qPCR in LMP2 and TAP2 knockdown (KD) cells. **Supplementary Fig. 3** Gelatin zymography was used for the determination of matrix metalloproteinase-2 (MMP-2) and MMP-9 activity in LMP2 and TAP2 OE cells. **Supplementary Fig. 4** Western blot analysis for the protein abundances of Wnt1, Wnt4, β-catenin, Cyclin D1 and c-Myc in the tumor tissues of LMP2 and TAP2 KD mice.

Supplementary Material 2

## Data Availability

The datasets generated during the current study are available from the corresponding author on reasonable request.
