# Supplementary material for: LMP2 and TAP2 impair tumor growth and metastasis by inhibiting Wnt/β-catenin signaling pathway and EMT in cervical cancer
Source: BMC Cancer. 2023 Nov 20;23:1128. doi: 10.1186/s12885-023-11639-y (PMC10662702; doi:10.1186/s12885-023-11639-y)
Supplement: Supplementary file 1 — Supplemental figures: Supplementary Fig. 1 The relative mRNA levels of Lmp2 and Tap2 were determined by RT-qPCR in LMP2 and TAP2 overexpression (OE) cells. Supplementary Fig. 2 The relative mRNA levels of Lmp2 and Tap2 were determined by RT-qPCR in LMP2 and TAP2 knockdown (KD) cells. Supplementary Fig. 3 Gelatin zymography was used for the determination of matrix metalloproteinase-2 (MMP-2) and MMP-9 activity in LMP2 and TAP2 OE cells. Supplementary Fig. 4 Western blot analysis for the protein abundances of Wnt1, Wnt4, β-catenin, Cyclin D1 and c-Myc in the tumor tissues of LMP2 and TAP2 KD mice. [file 12885_2023_11639_MOESM1_ESM.pdf]

Supplementary information

LMP2 and TAP2 impair tumor growth and metastasis by inhibiting Wnt/β-catenin signaling pathway and EMT in cervical cancer

Zhengyan Cheng, Hongbo Wang, Zewei Yang, Jiaxu Li and Xing Chen

Methods

Real-time quantitative RT-PCR (RT-qPCR)

To confirm the effects of LMP2 and TAP2 on cervical cell proliferation and migration, Hela cells in 12-well plates were transfected with LMP2 and TAP2 expression plasmids for 24 and 48 h. Total RNA was extracted from the treated cells with TRIzol reagent (Invitrogen), followed by first-strand cDNA synthesis. Each quantitative real-time PCR (qPCR) experiment was performed at least three times and was conducted via the SYBR green PCR assay (Applied Biosystems) using the cDNA described above as the template. The results are expressed as the relative gene expression level with normalization to the expression level of glyceraldehyde-3-phosphate dehydrogenase (GAPDH). All primers utilized here were designed at National Center for Biotechnology Information (NCBI) and as follows: *Lmp2* forward, 5'- CTCCGGCAGCACCTTTATCT-3' and reverse, 5'-TGCCCAAGATGACTCGATGG-3'; *Tap2* forward, 5'-CCTGGCCGAGCGTAGC-3' and reverse, 5'-AGCCCTTGAGGAAGCAAAGT-3'; *Gapdh* forward, 5'-GACAGTCAGCCGCATCTTCT-3' and reverse, 5'-GCGCCCAATACGACCAAATC-3'.

Gelatin zymography

The proteins were separated in 7.5% polyacrylamide gels containing sodium dodecyl sulfate (SDS) and 1 mg/ml gelatin. After electrophoresis,

the gels were washed with 2.5% Triton X100 to remove the SDS and incubated overnight in 50 m mol L^{-1} Tris-HCl pH 7.5, 10 m mol L^{-1} $CaCl_2$ at 37°C for the development of zymolytic bands. To inhibit the activity of the metalloprotease during enzyme development, the gels were incubated with 10 m mol L^{-1} EDTA. Protease bands were detected by the absence of Coomassie Brilliant Blue staining of digested gelatin. The different protease bands were qualitatively and quantitatively analyzed with ImageJ software.

Supplemental figures

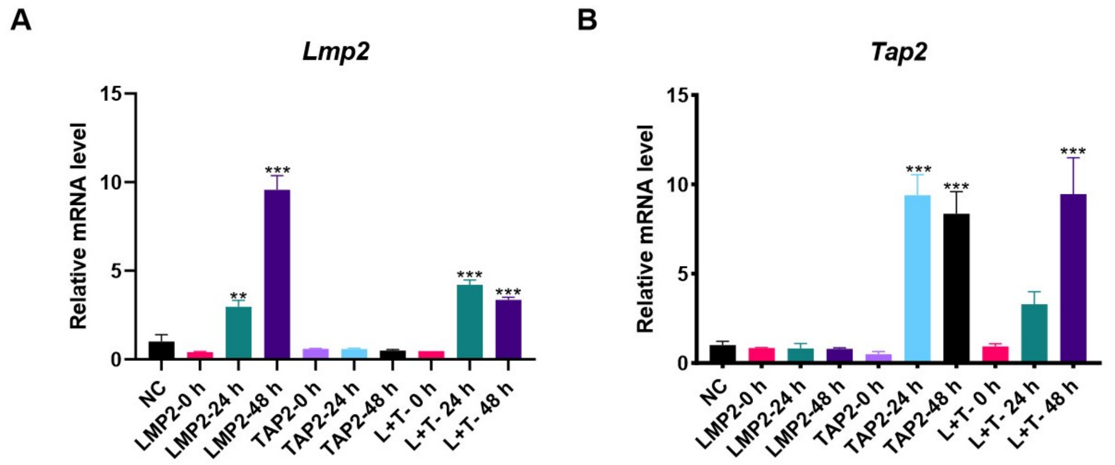

Supplementary Fig. 1 The relative mRNA levels of *Lmp2* and *Tap2* were determined by RT-qPCR in LMP2 and TAP2 overexpression (OE) cells.

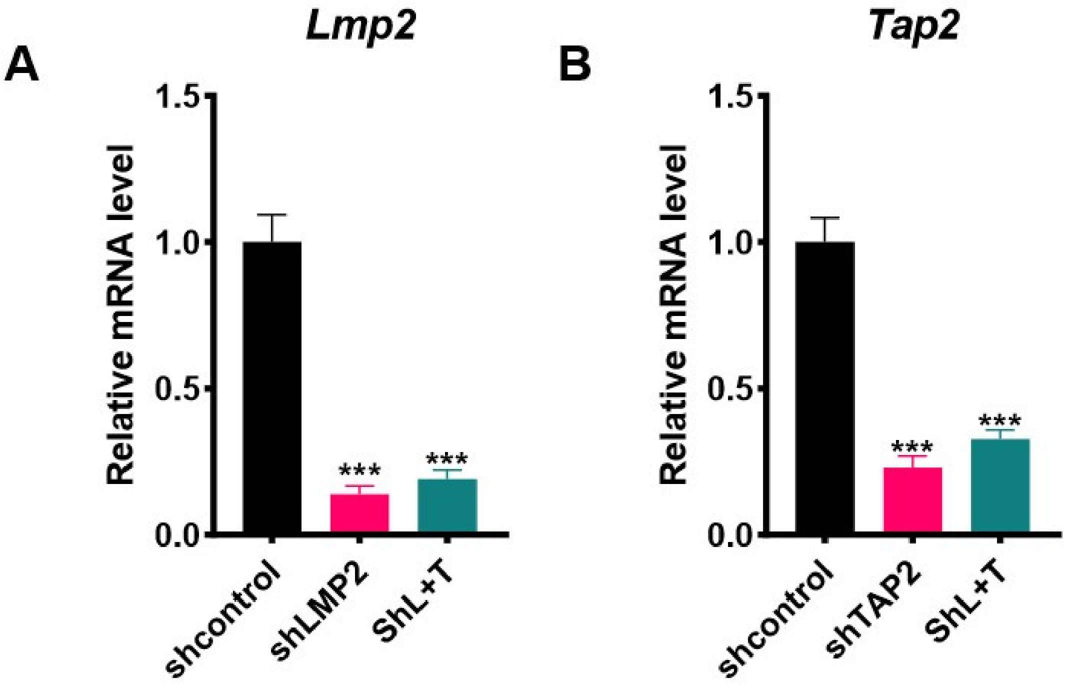

Supplementary Fig. 2 The relative mRNA levels of *Lmp2* and *Tap2* were determined by RT-qPCR in LMP2 and TAP2 knockdown (KD) cells.

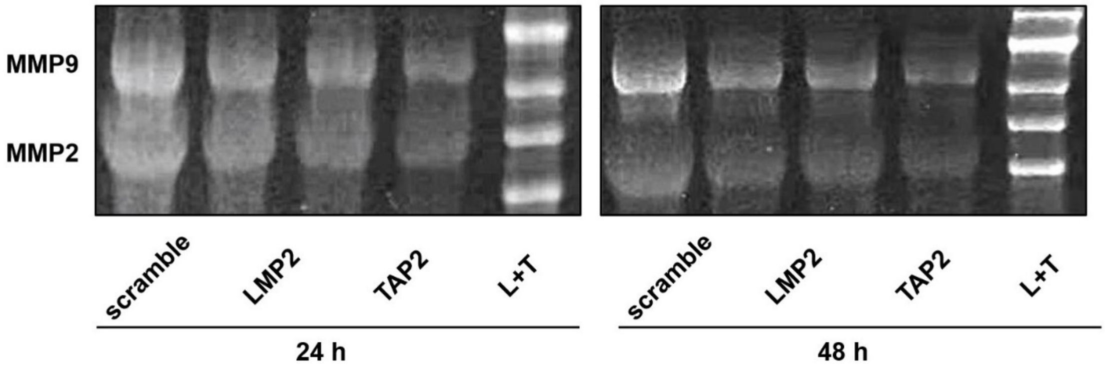

Supplementary Fig. 3 Gelatin zymography was used for the determination of matrix metalloproteinase-2 (MMP-2) and MMP-9 activity in LMP2 and TAP2 OE cells.

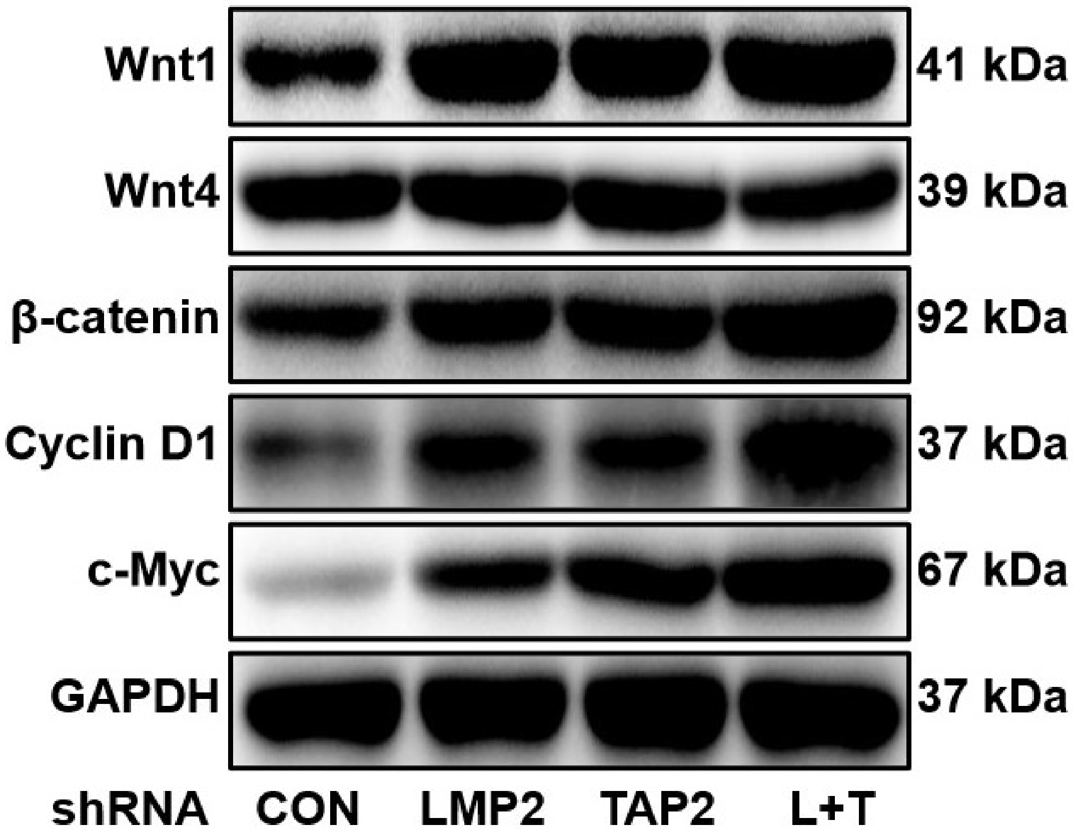

Supplementary Fig. 4 Western blot analysis for the protein abundances of Wnt1, Wnt4, β-catenin, Cyclin D1 and c-Myc in the tumor tissues of LMP2 and TAP2 KD mice.
