# Supplementary material for: LMP2 and TAP2 impair tumor growth and metastasis by inhibiting Wnt/β-catenin signaling pathway and EMT in cervical cancer
Source: BMC Cancer. 2023 Nov 20;23:1128. doi: 10.1186/s12885-023-11639-y (PMC10662702; doi:10.1186/s12885-023-11639-y)

**Supplementary information**

**LMP2 and TAP2 impair tumor growth and metastasis by inhibiting Wnt/β-catenin signaling pathway and EMT in cervical cancer**

**Zhengyan Cheng, Hongbo Wang, Zewei Yang, Jiaxu Li and Xing Chen**

**Origin images of WB-1**

**Figure 1A**

LMP2


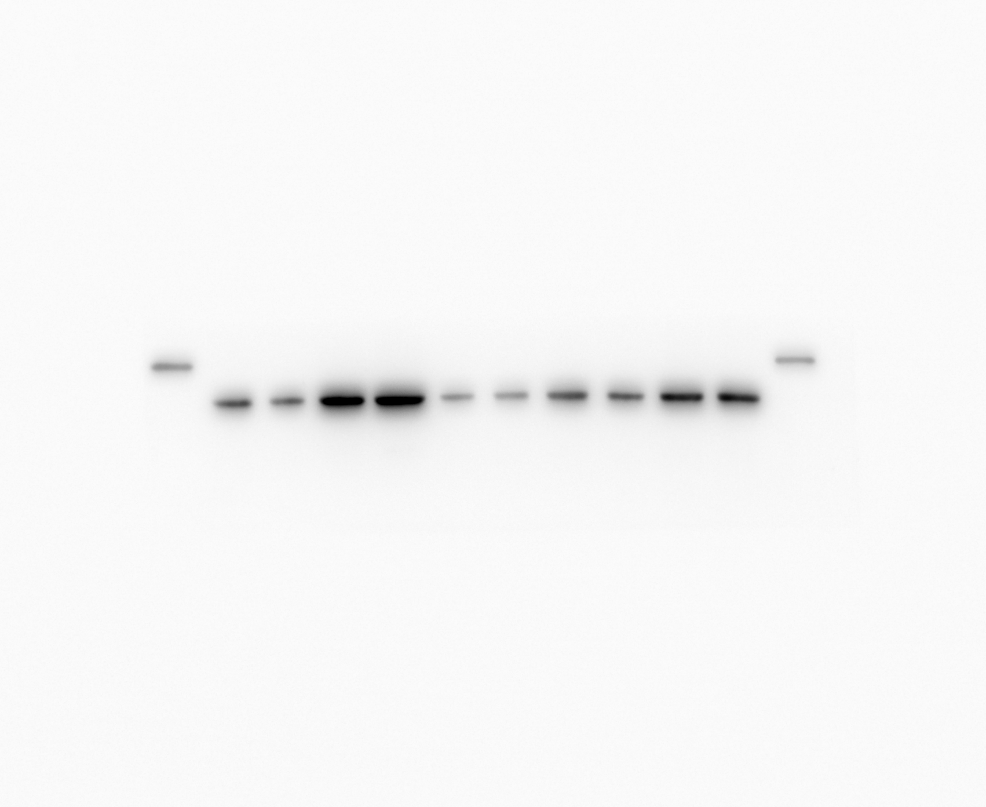


TAP2


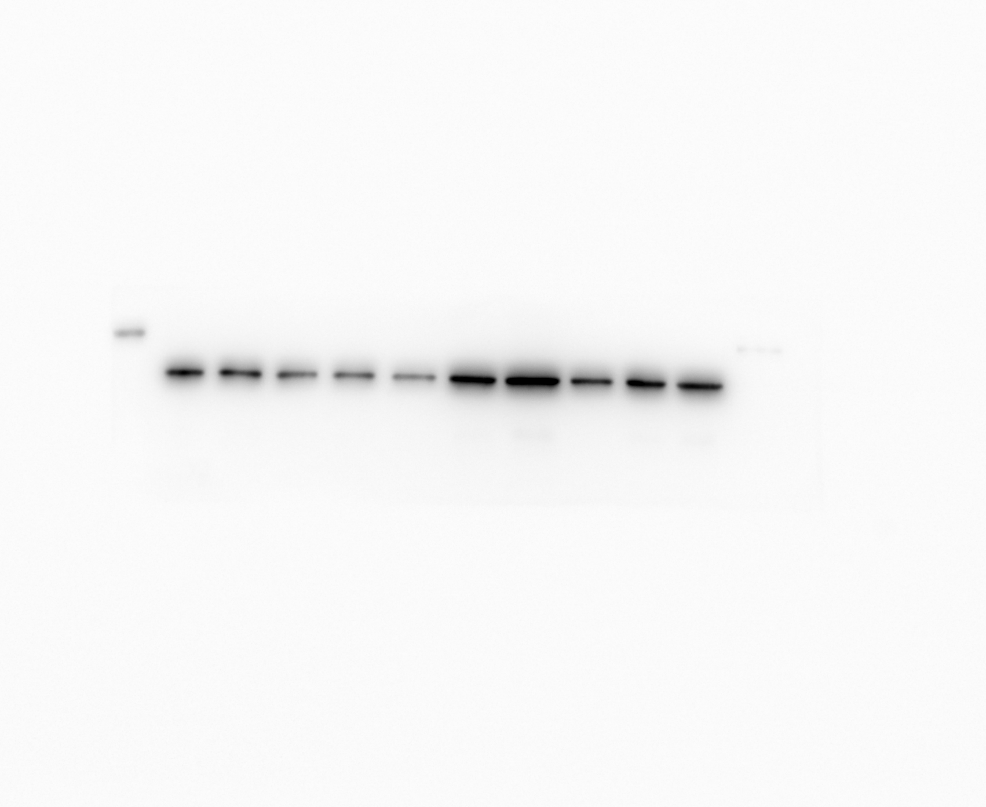


GAPDH


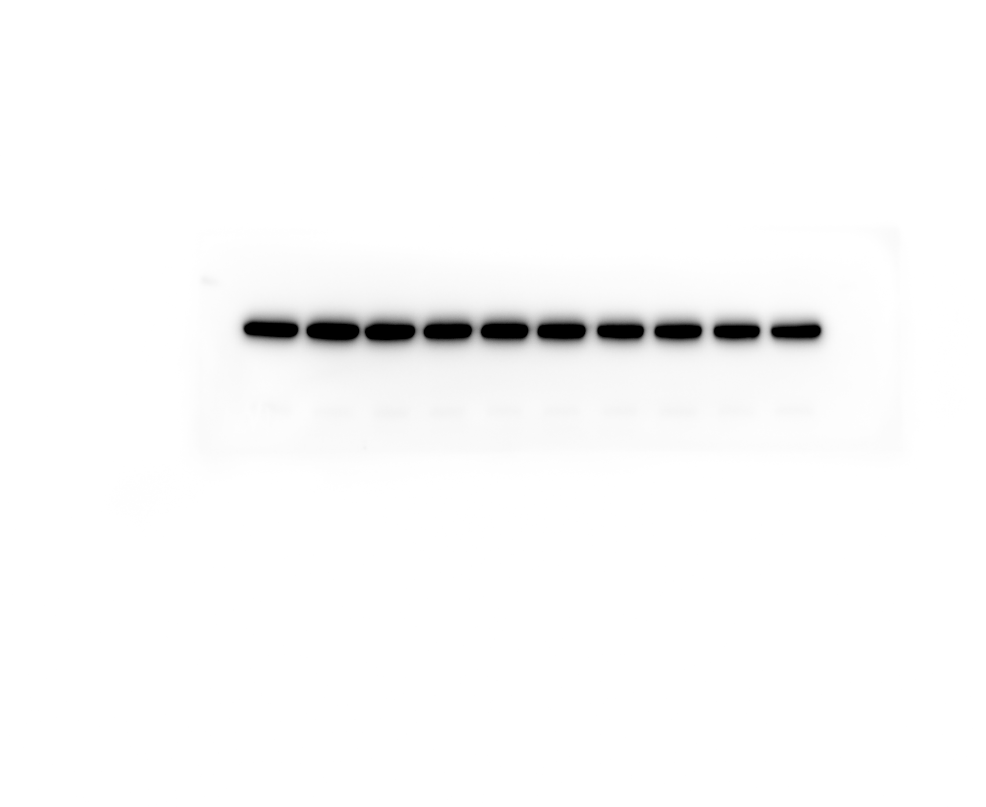


**Figure 1E**

LMP2


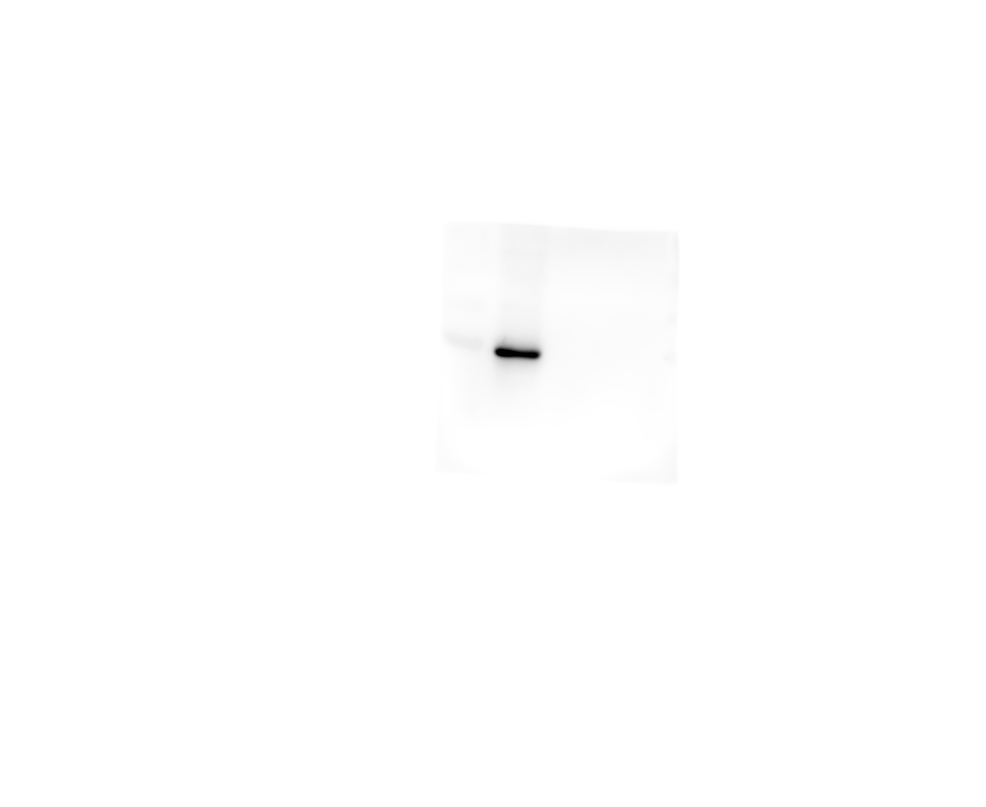


GAPDH


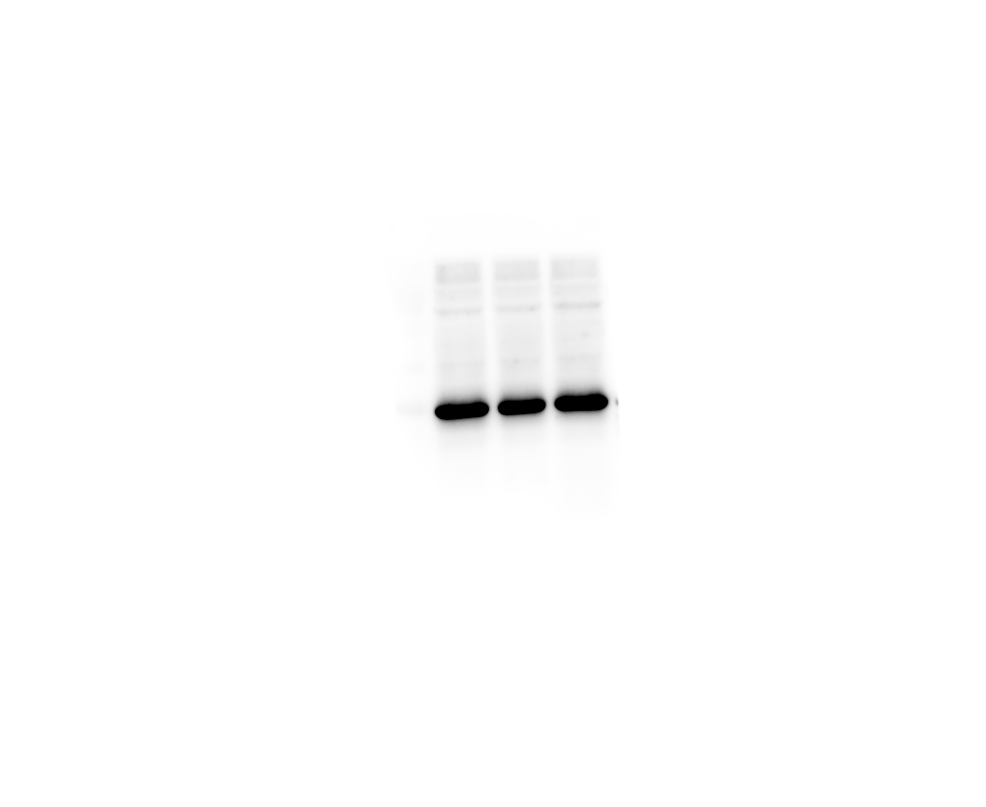


TAP2


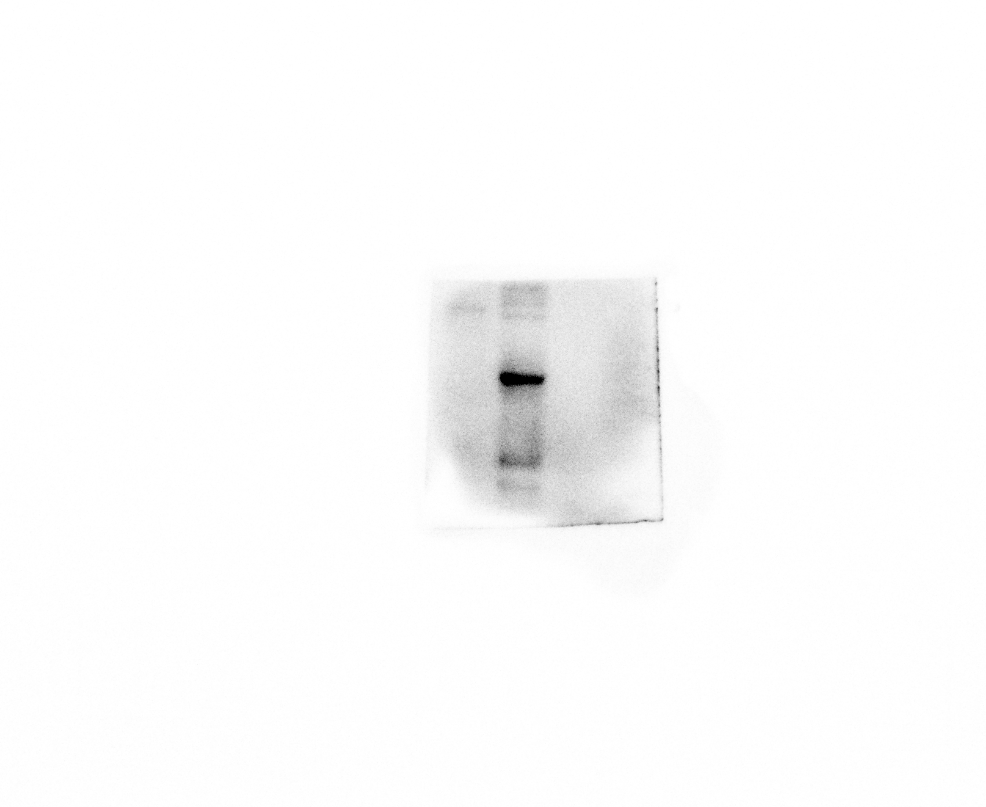


GAPDH


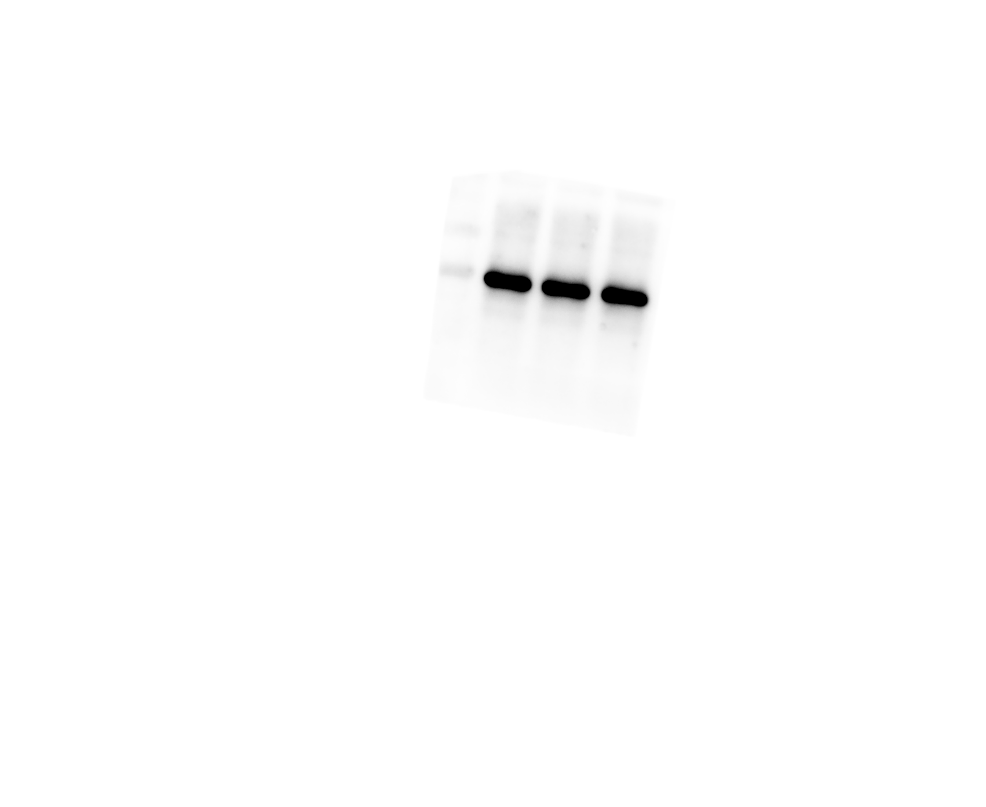


**Figure 3A**

MMP2 (24 h)


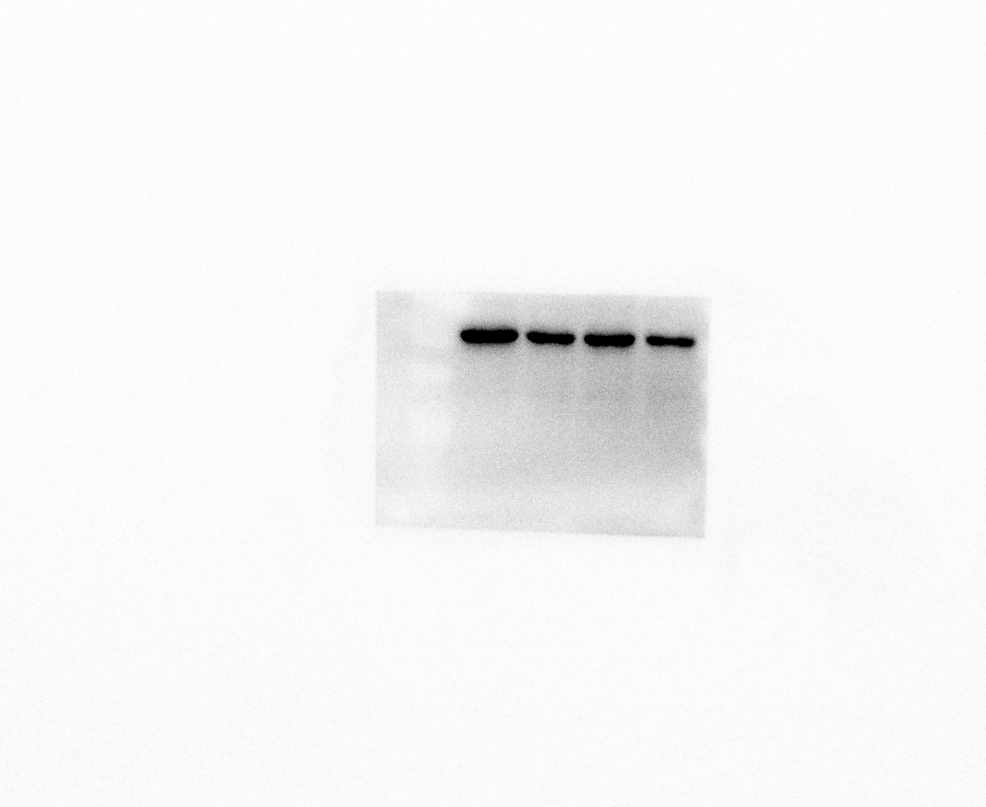


MMP9 (24 h)


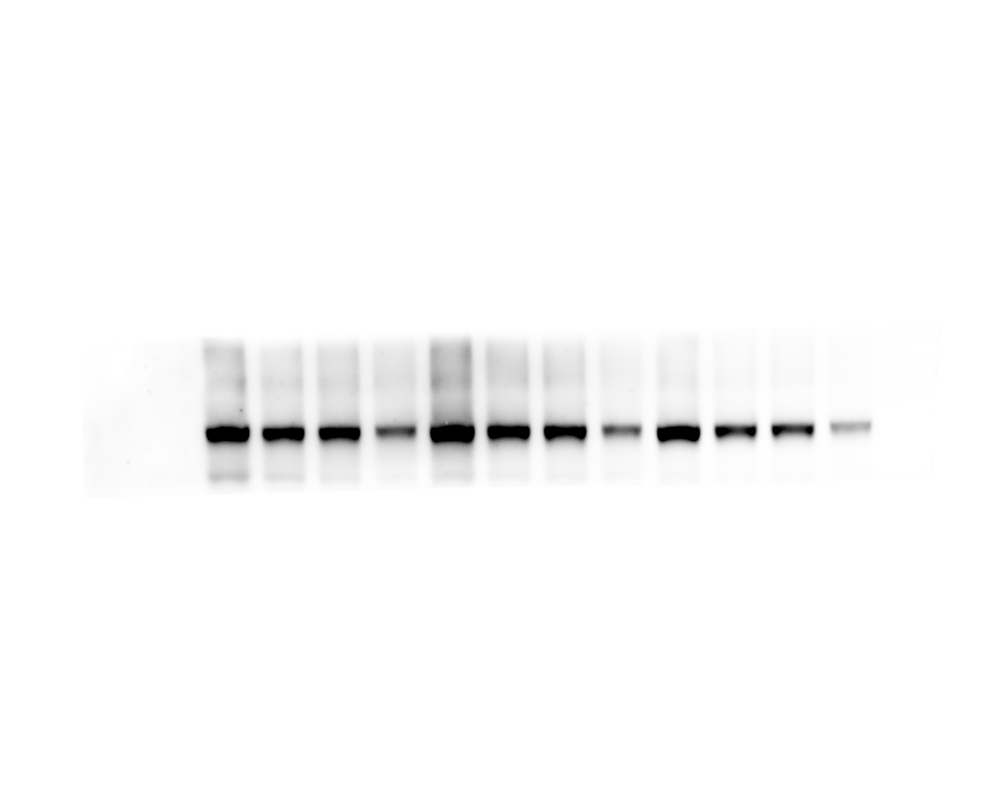


Vimentin (24 h)


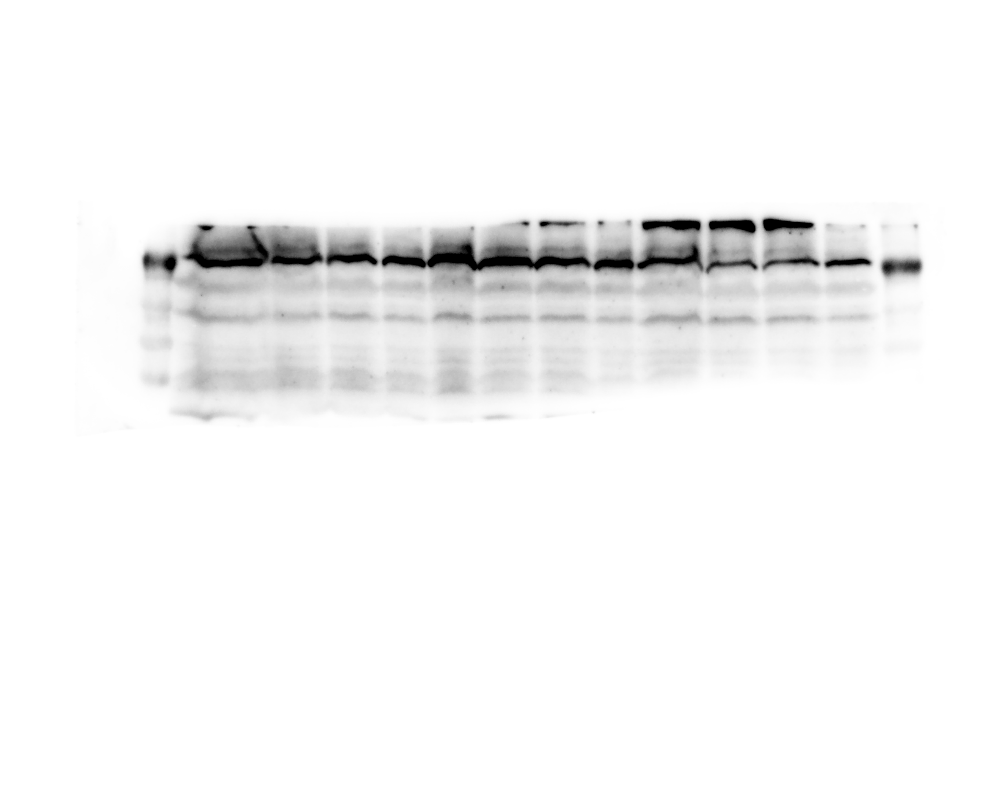


β-Tubulin (24 h)


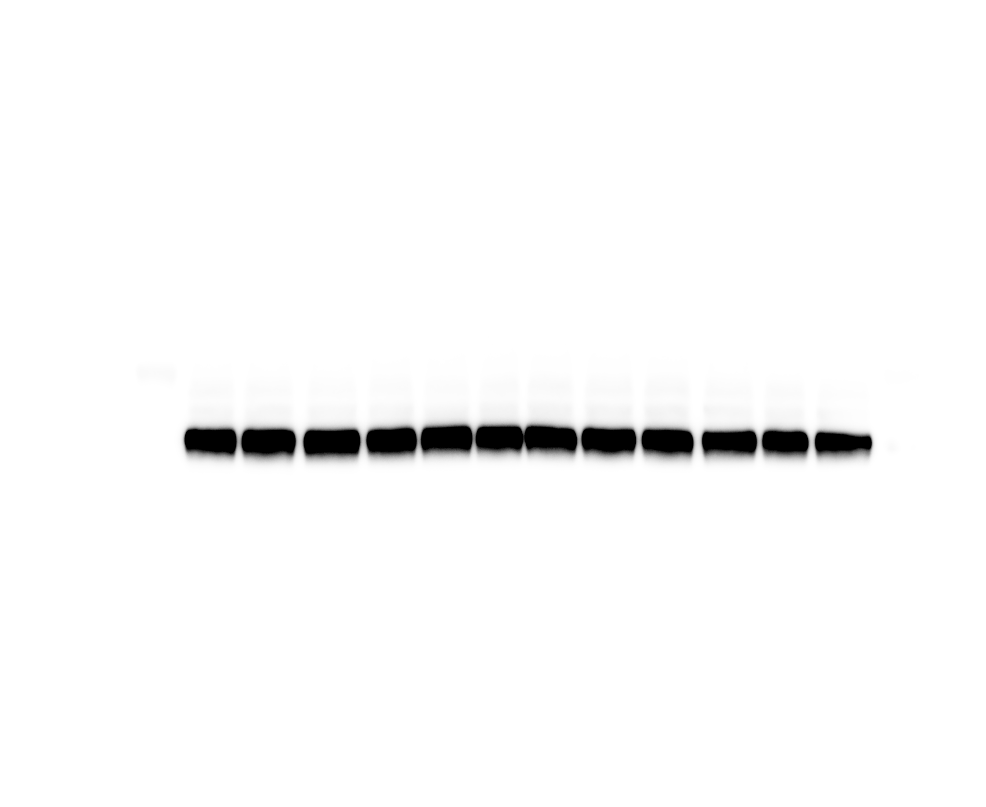


MMP2 (48 h)


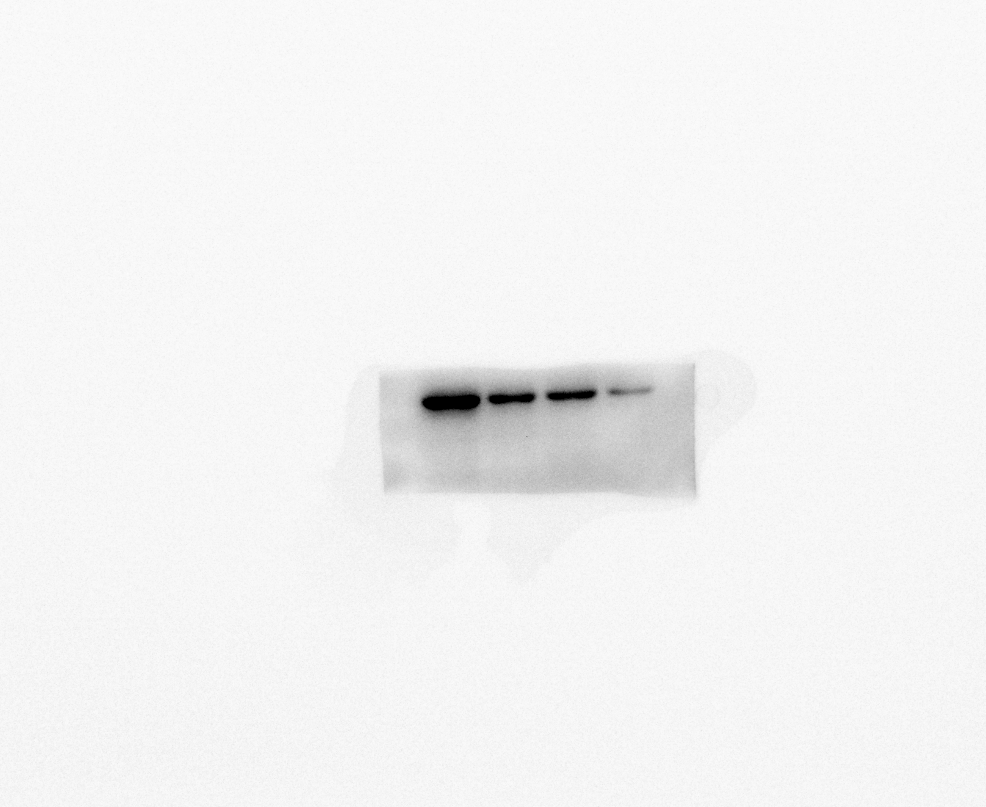


MMP9 (48 h)


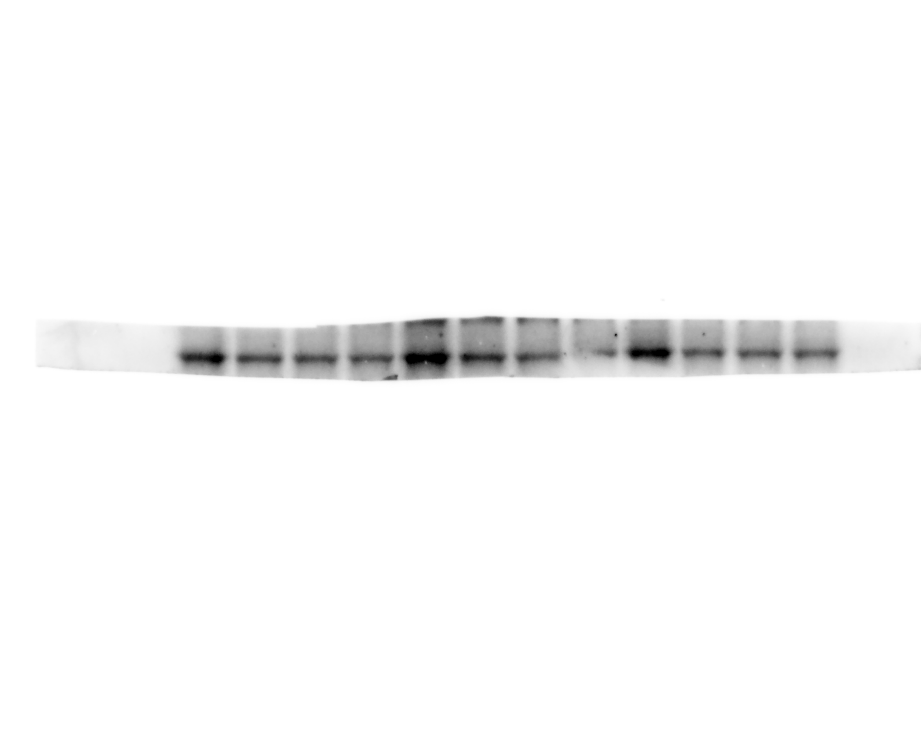


Vimentin (48 h)


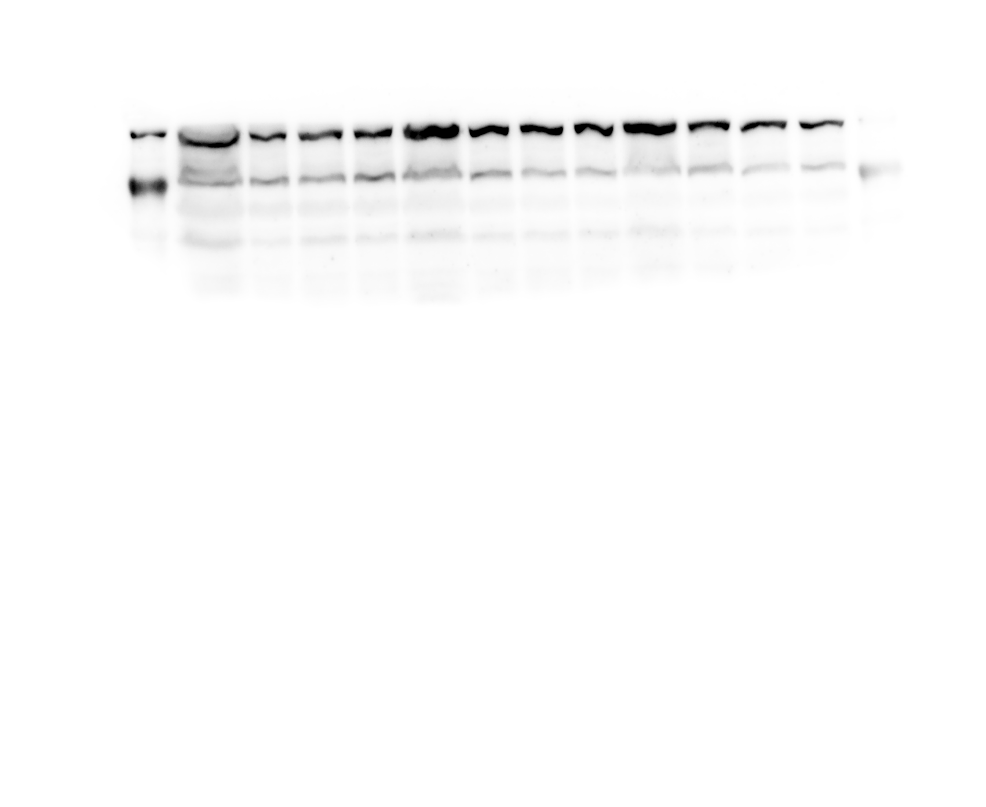


β-Tubulin (48 h)


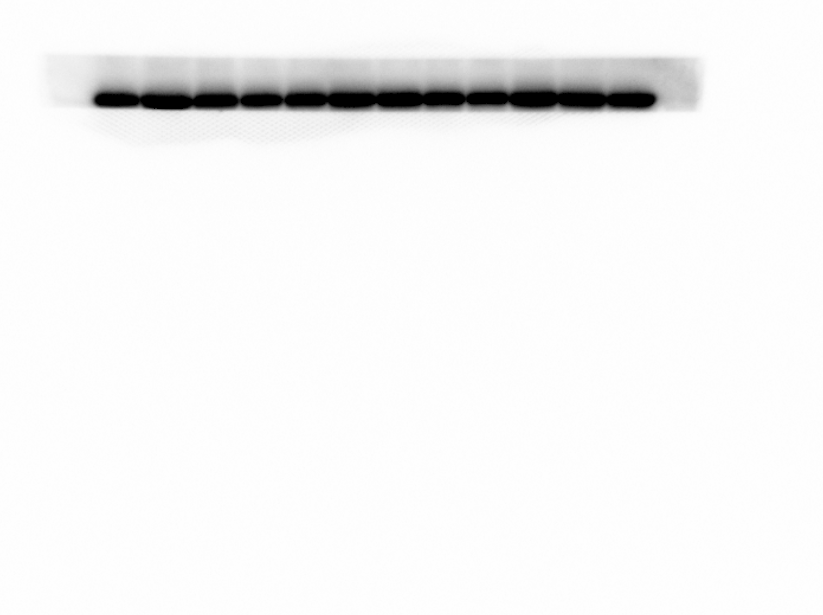


**Figure 3C**

E-cadherin (24 h)


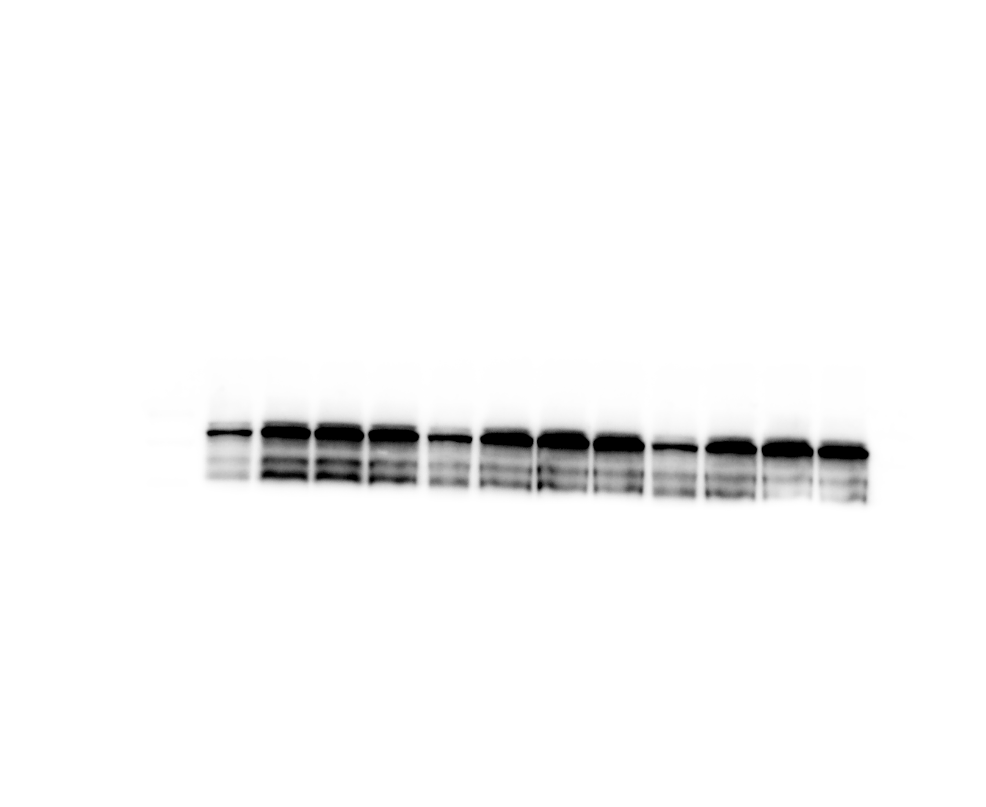


N-cadherin (48 h)


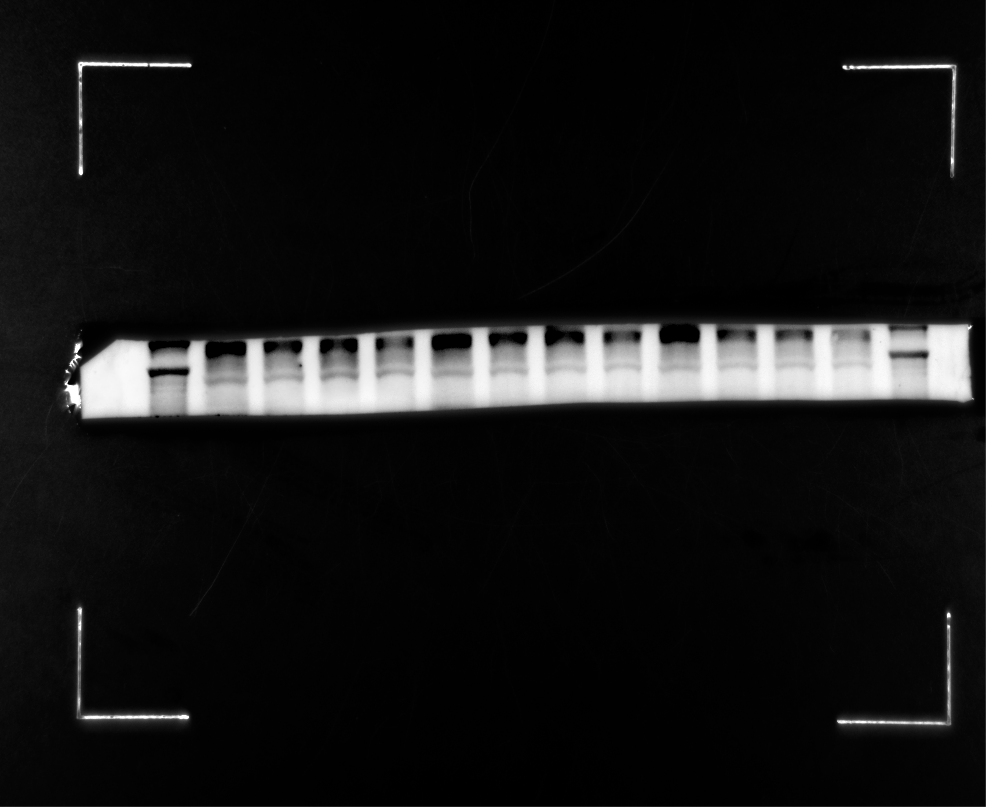


β-Tubulin (24 h)


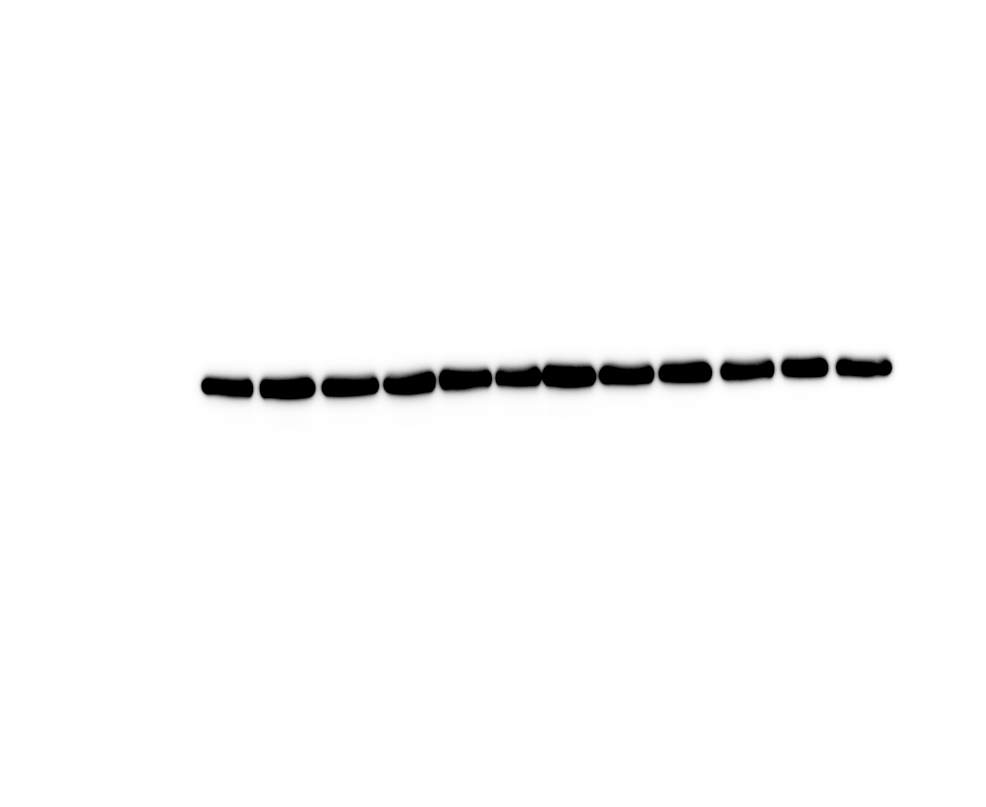


E-cadherin (48 h)


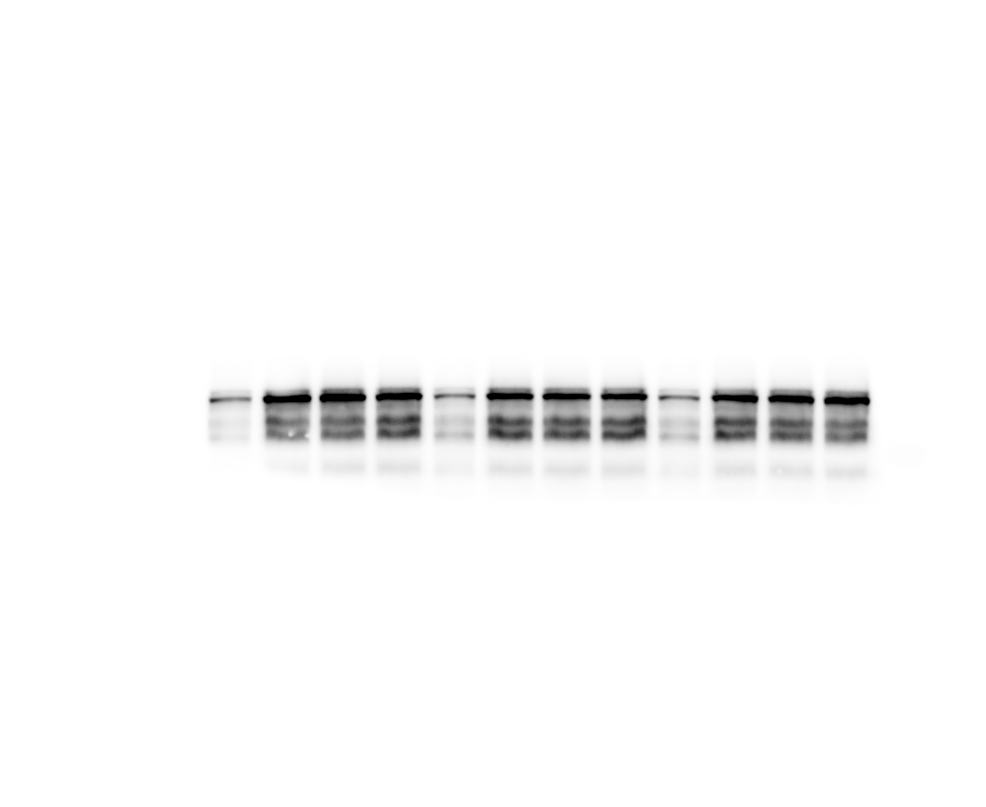


N-cadherin (48 h)


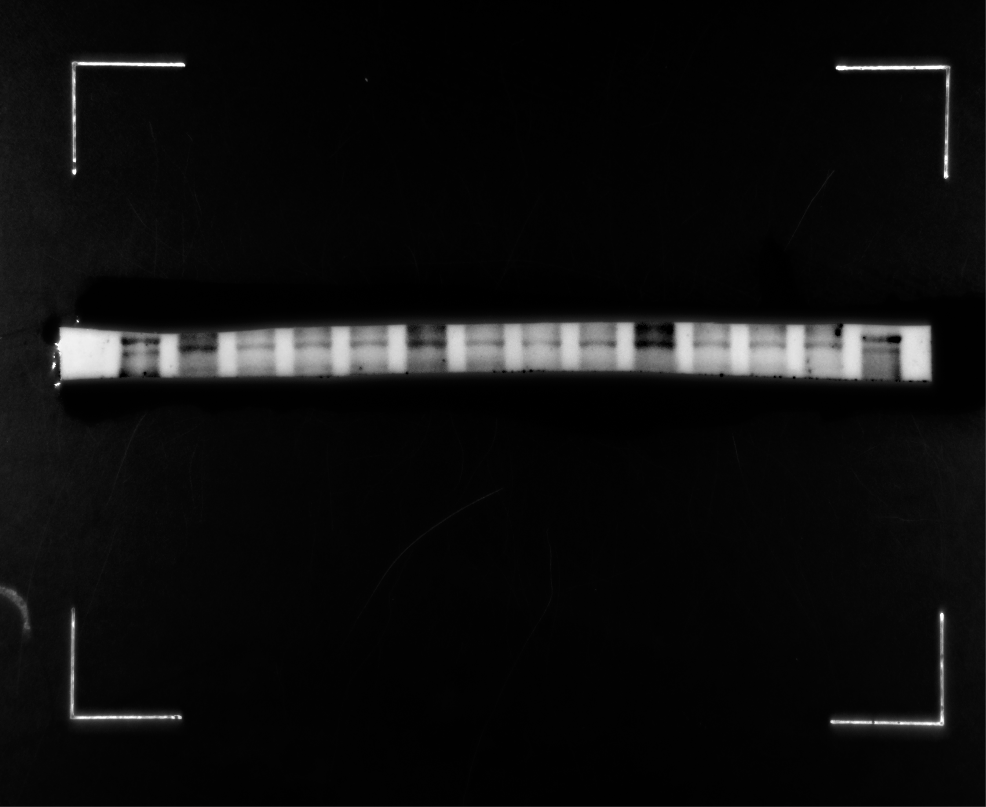


β-Tubulin (48 h)


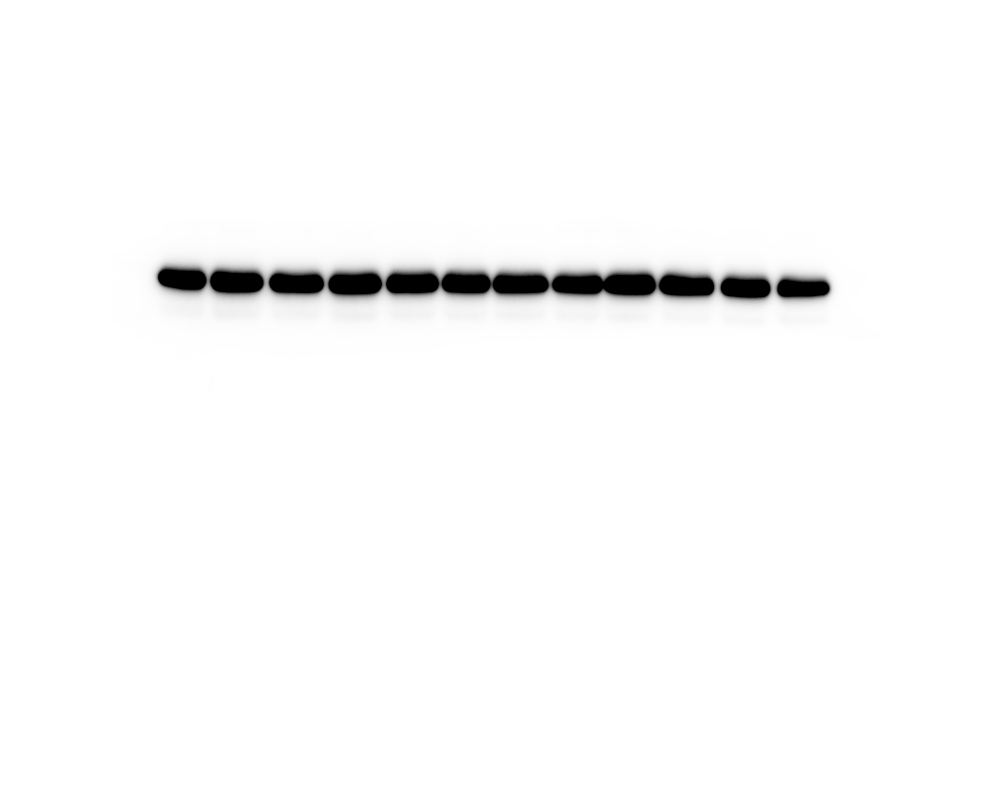


**Figure 4A**

Wnt1 (24 h)


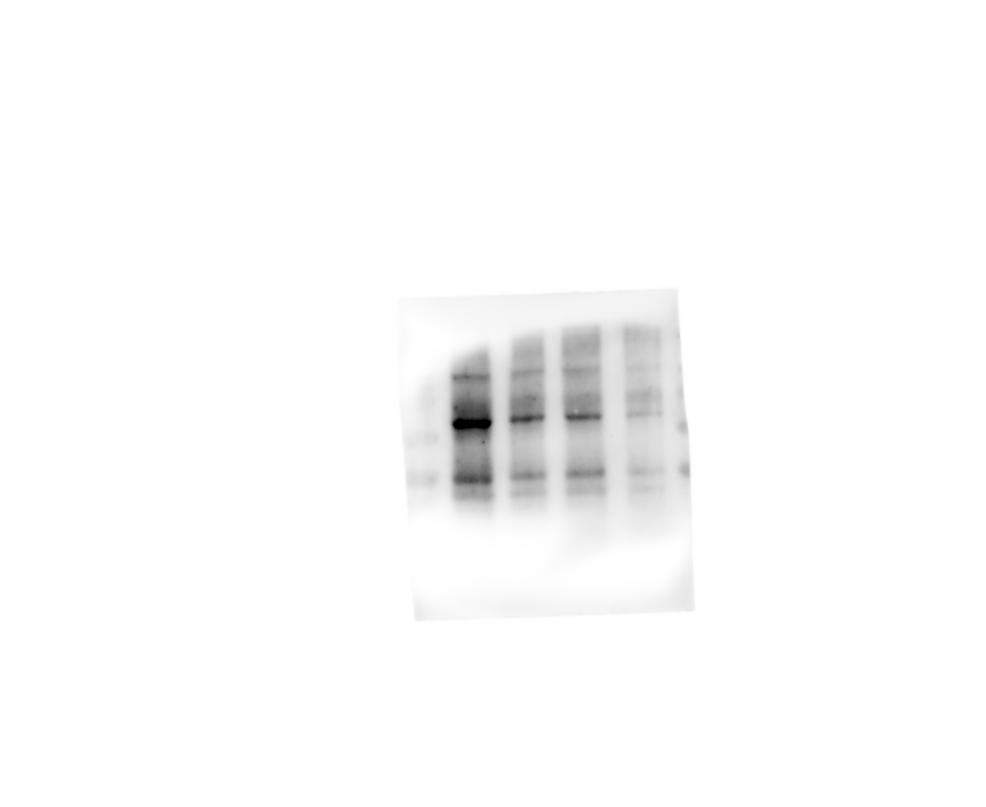


Wnt4 (24 h)


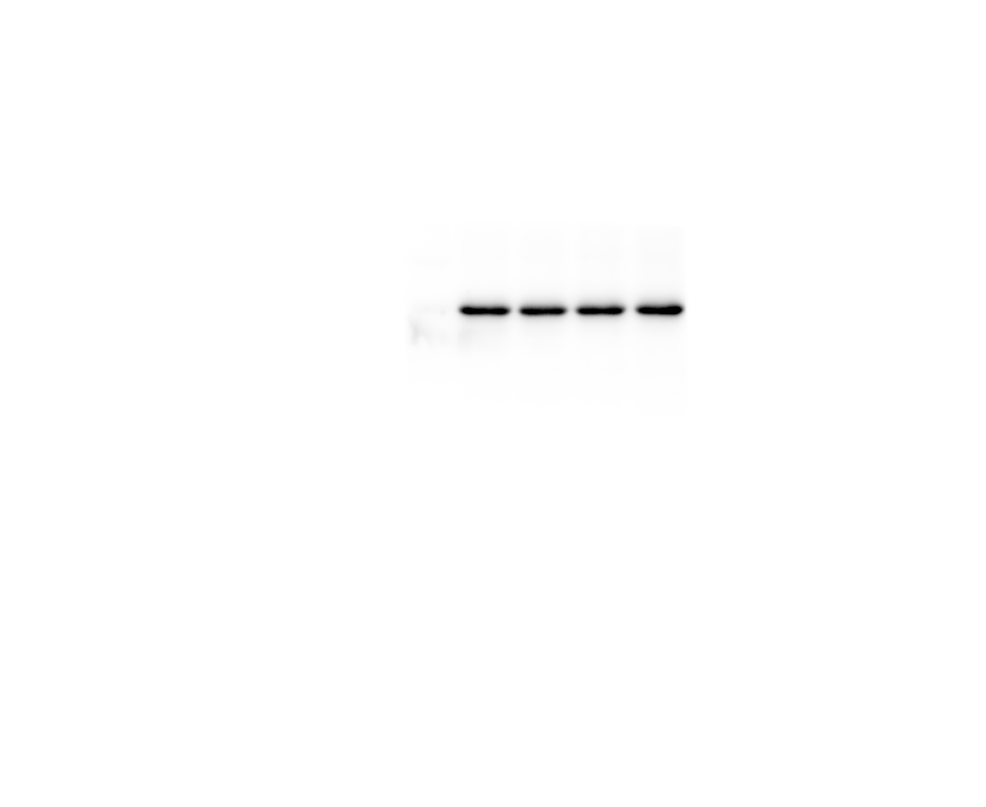


β-catenin (24 h)


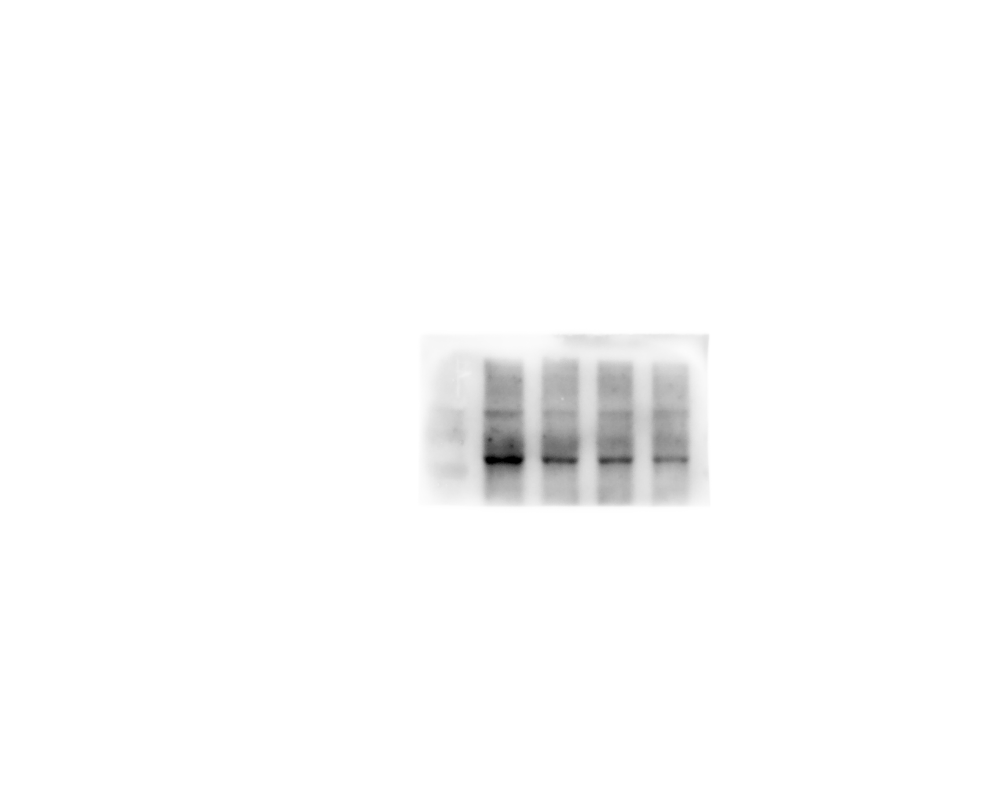


Cyclin D1 (24 h)


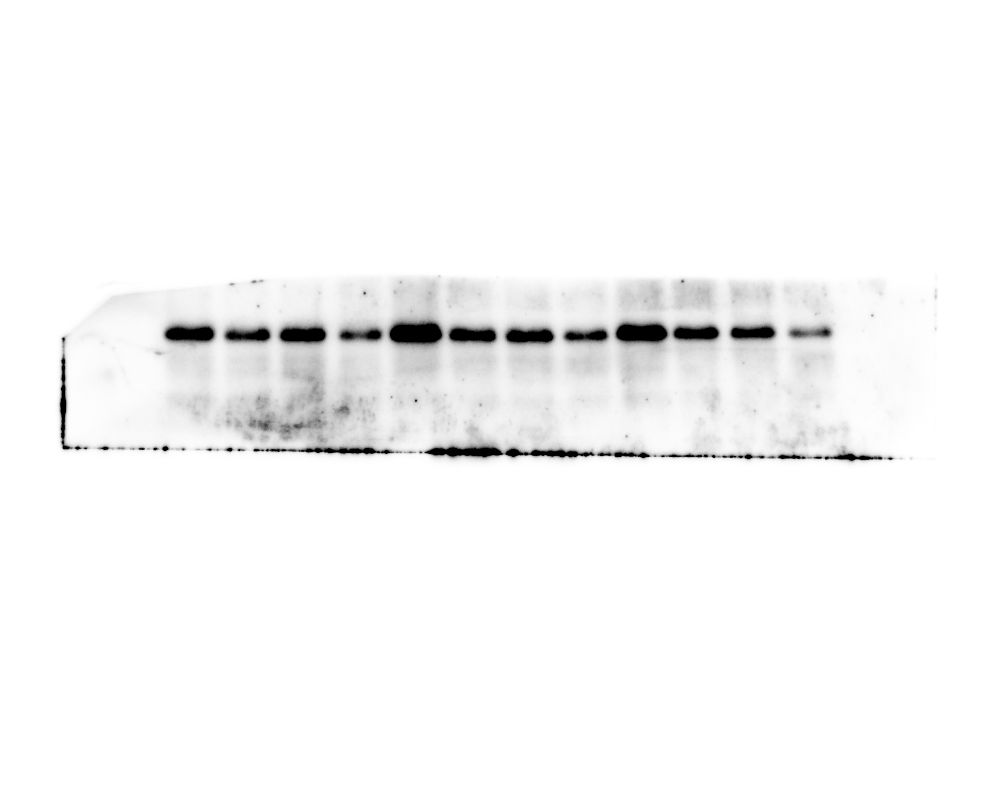


c-Myc (24 h)


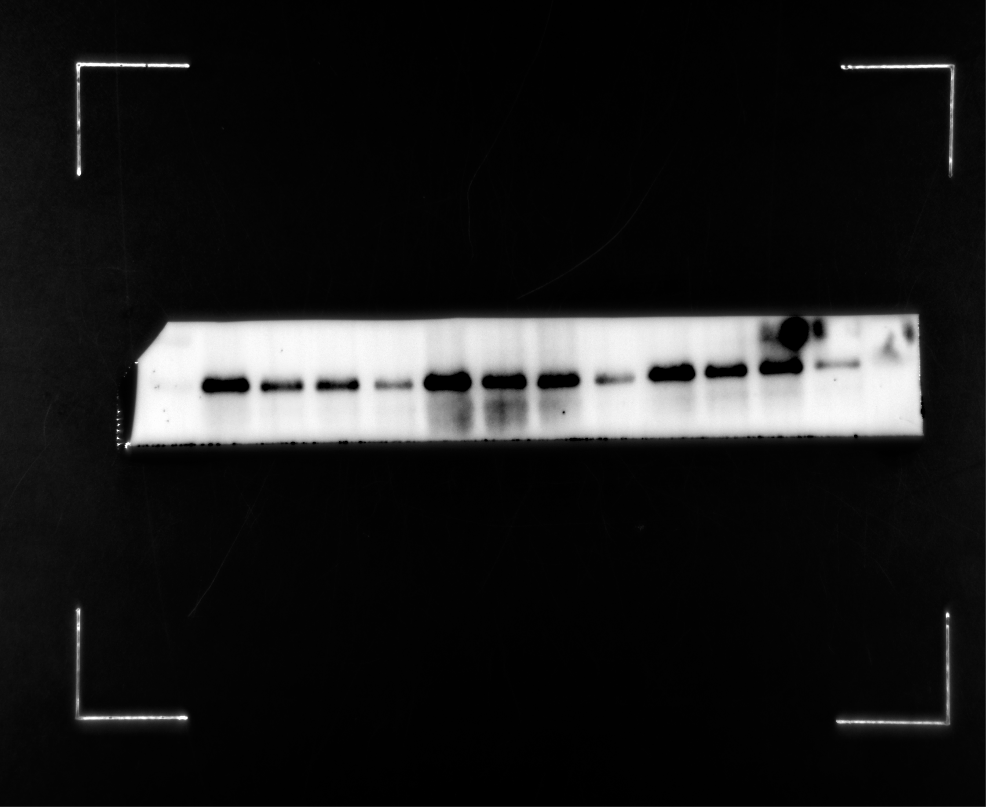


β-Tubulin (24 h)


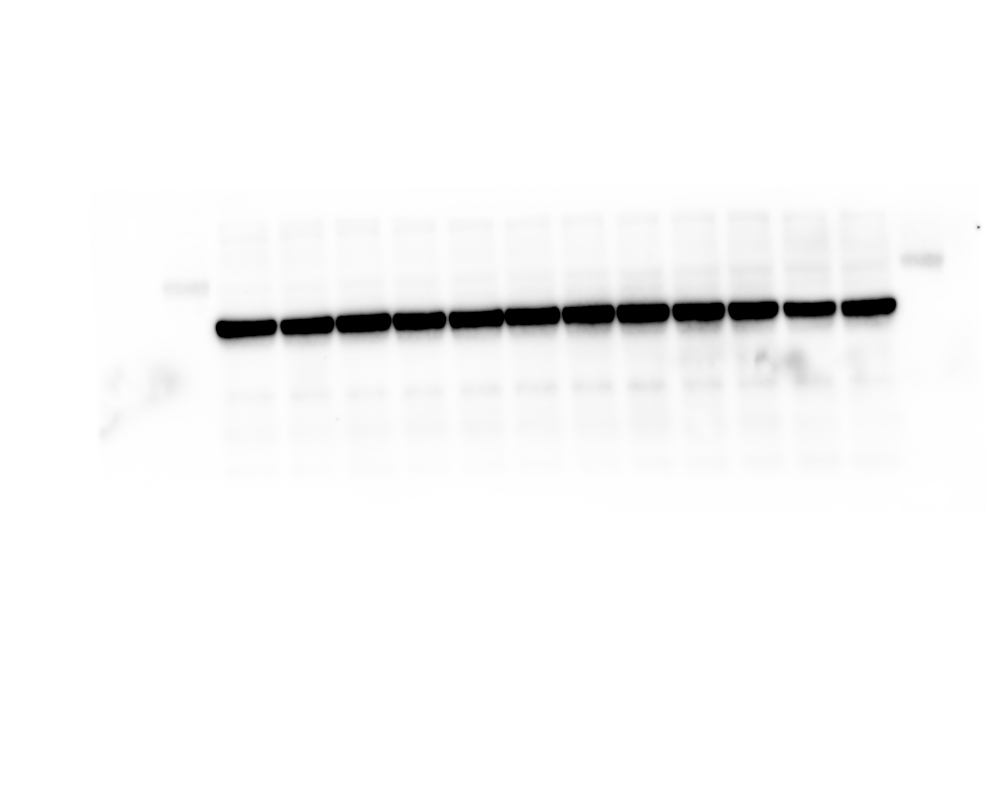


Wnt1 (48 h)


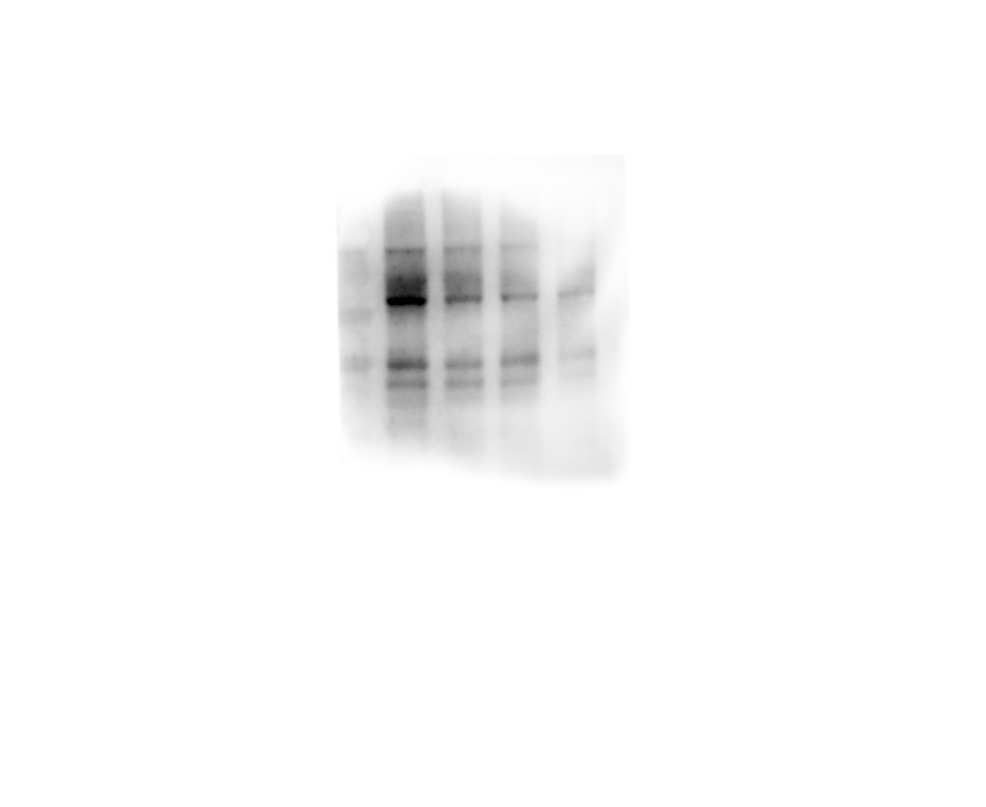


Wnt4 (48 h)


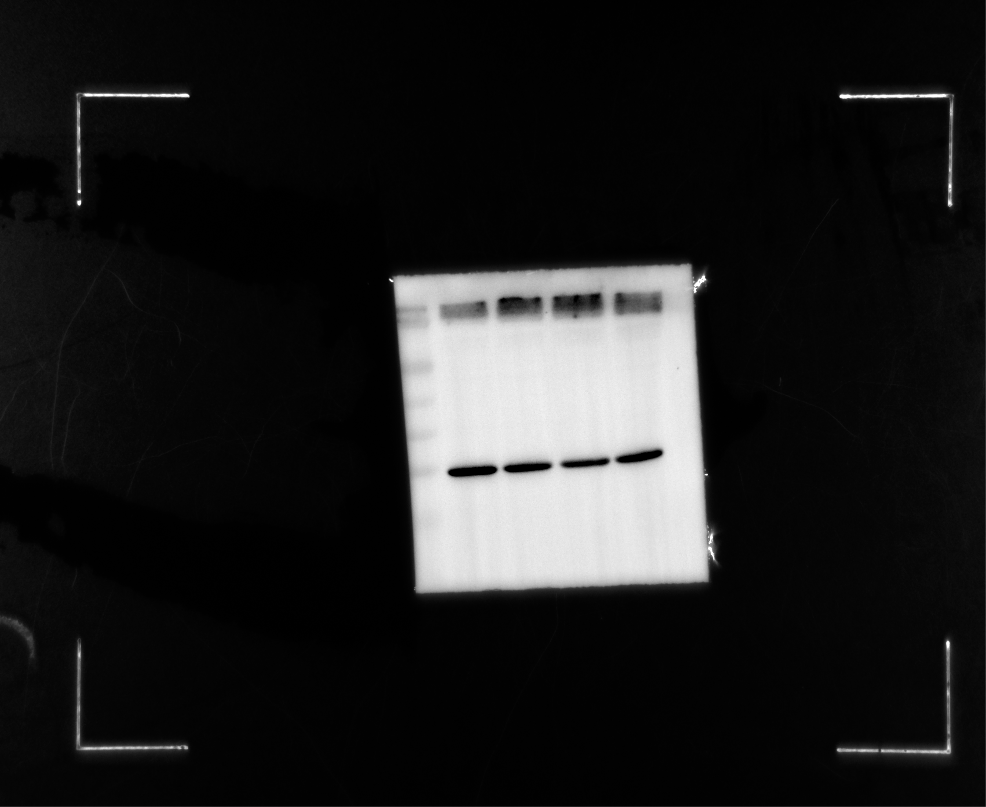


β-catenin (48 h)


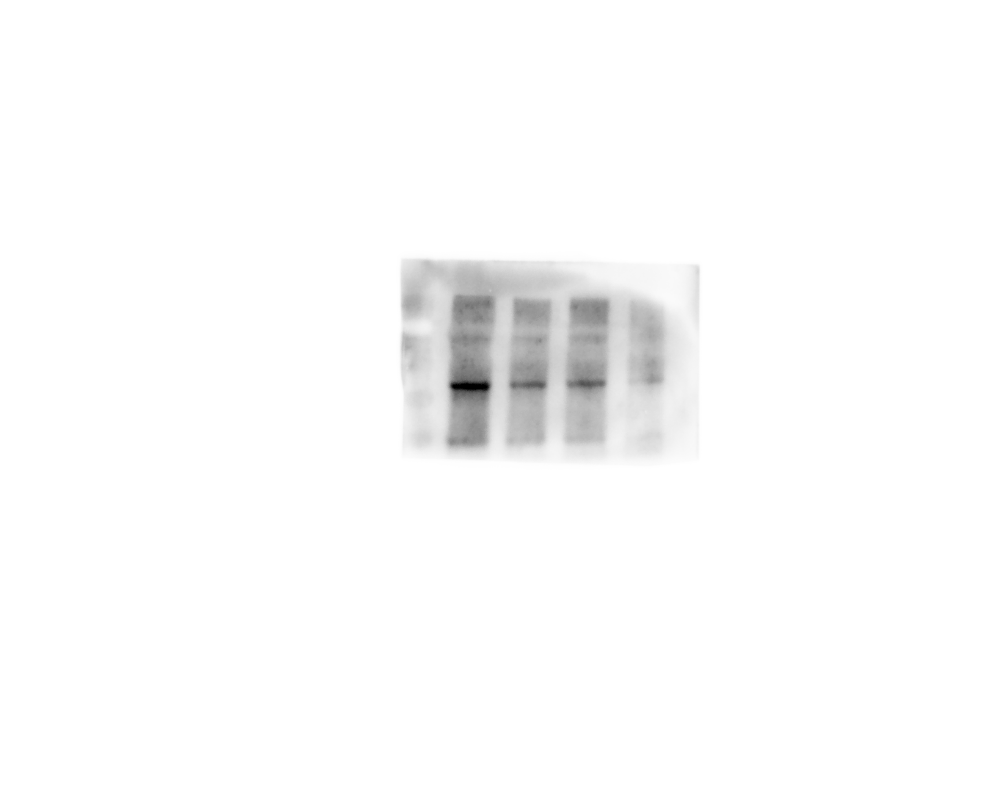


Cyclin D1 (48 h)


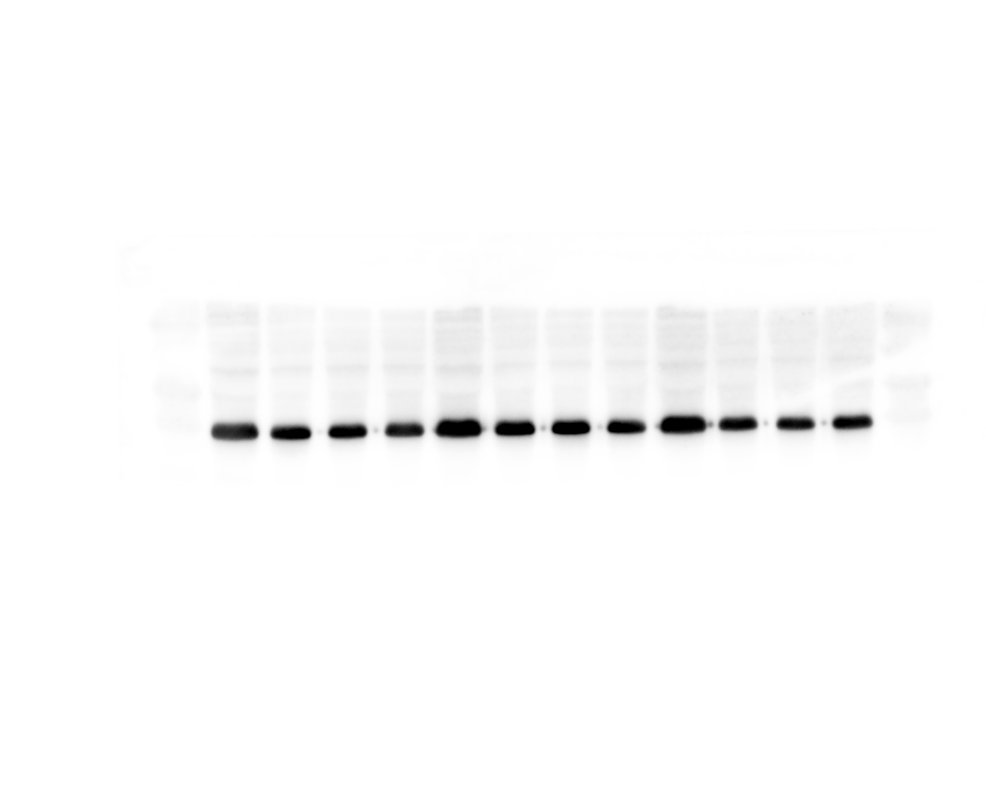


c-Myc (48 h)


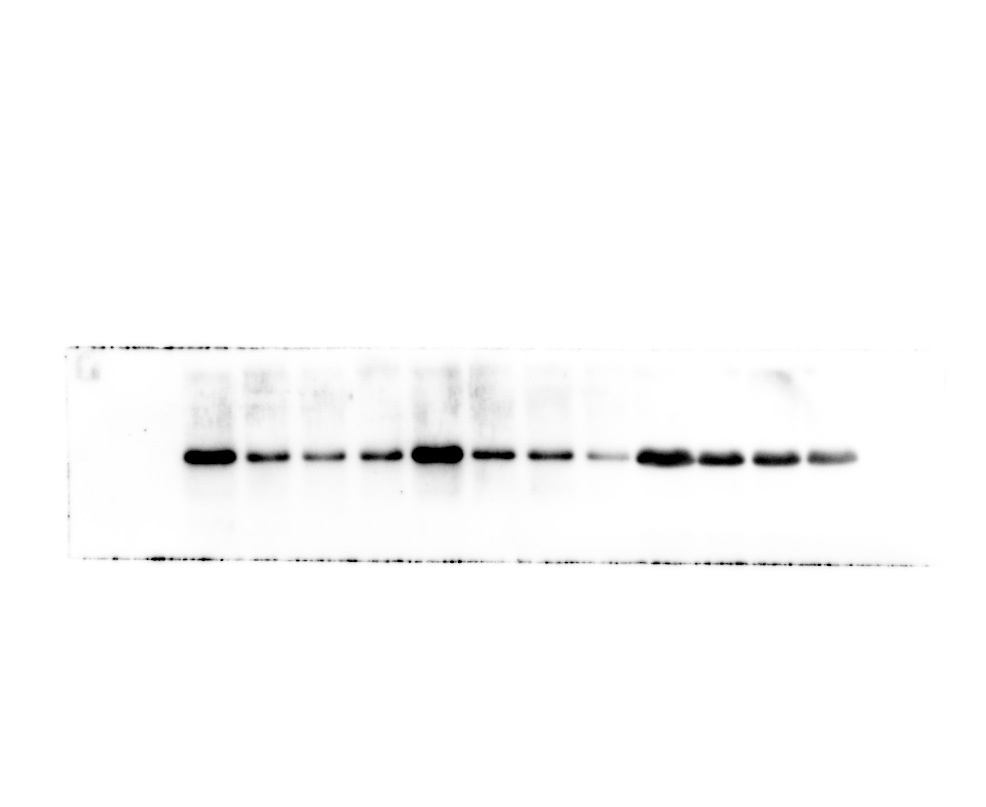


β-Tubulin (48 h)


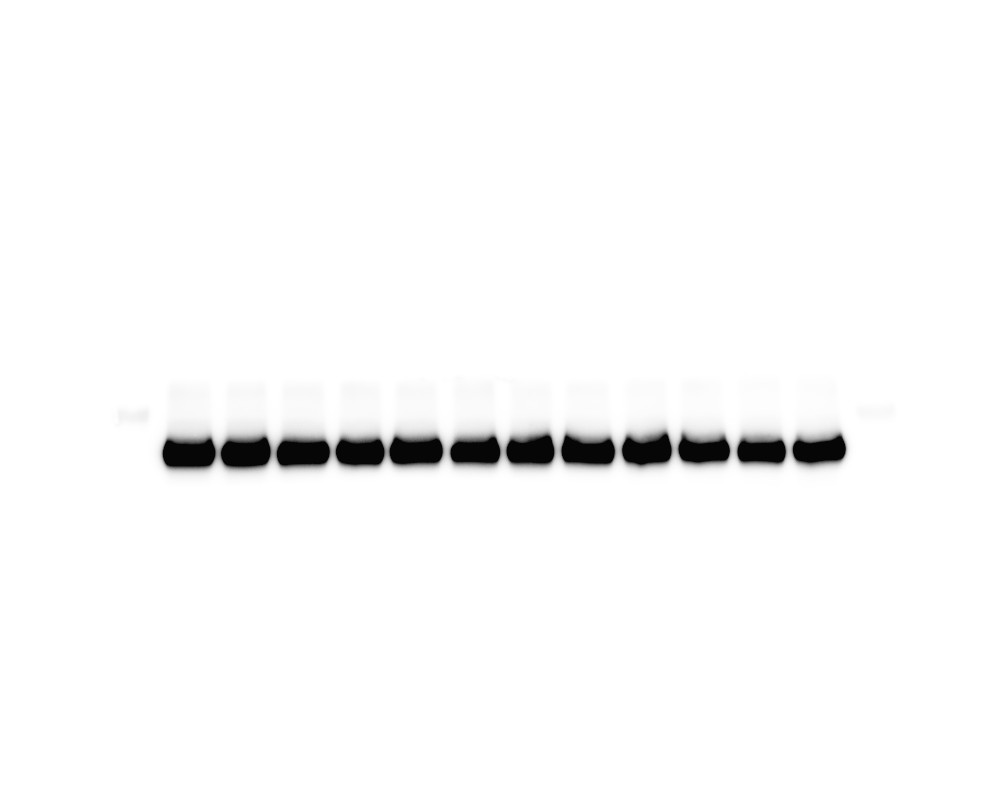


**Figure 4C**

Wnt1


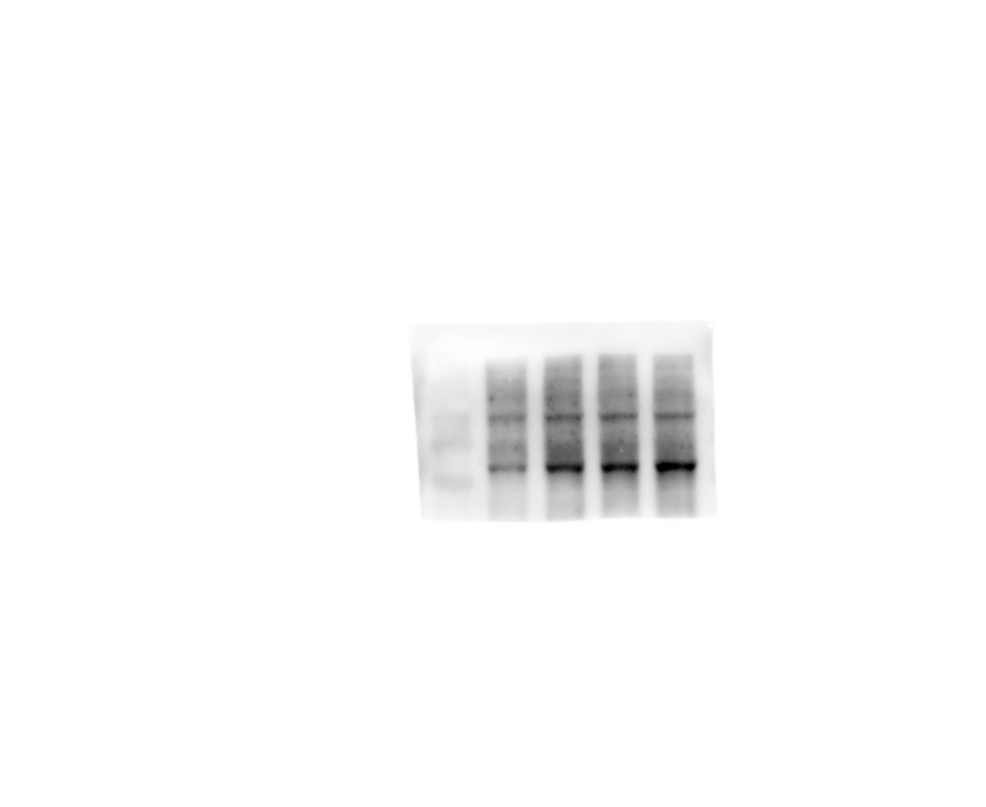


Wnt4


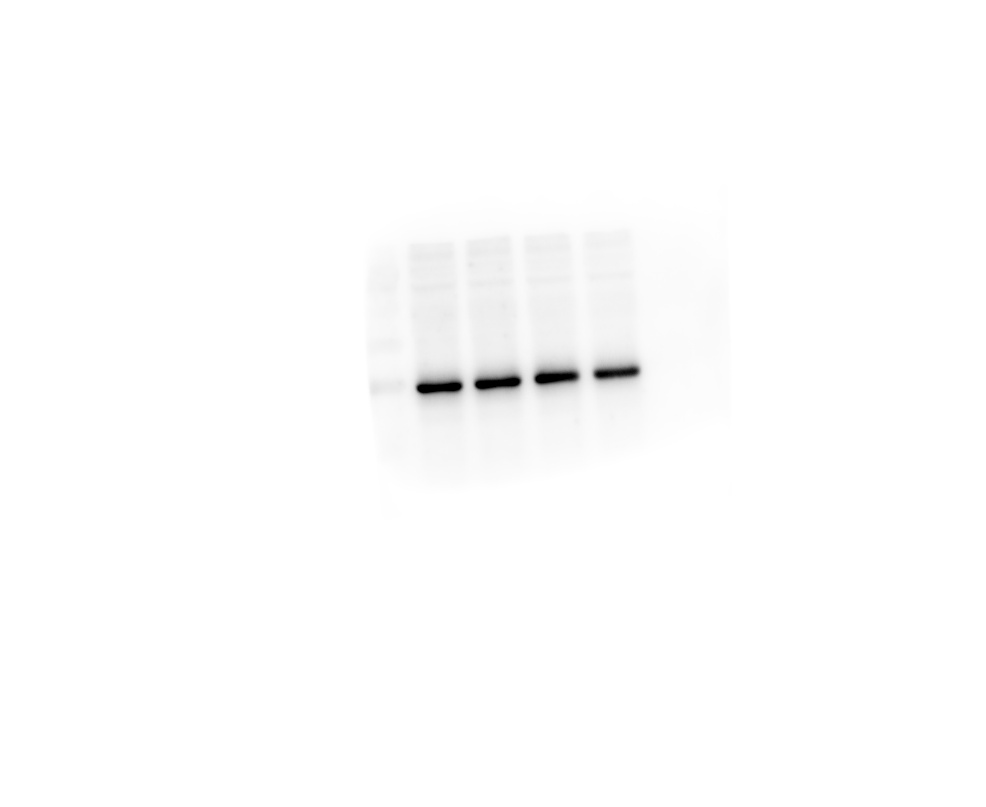


β-catenin


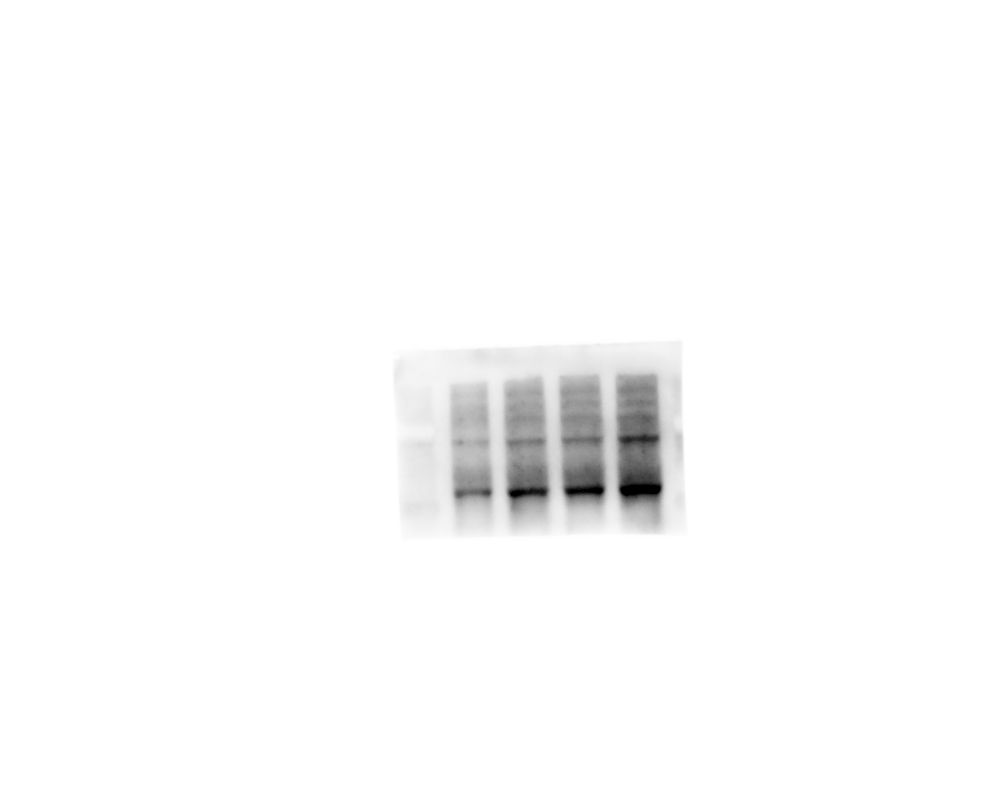


Cyclin D1


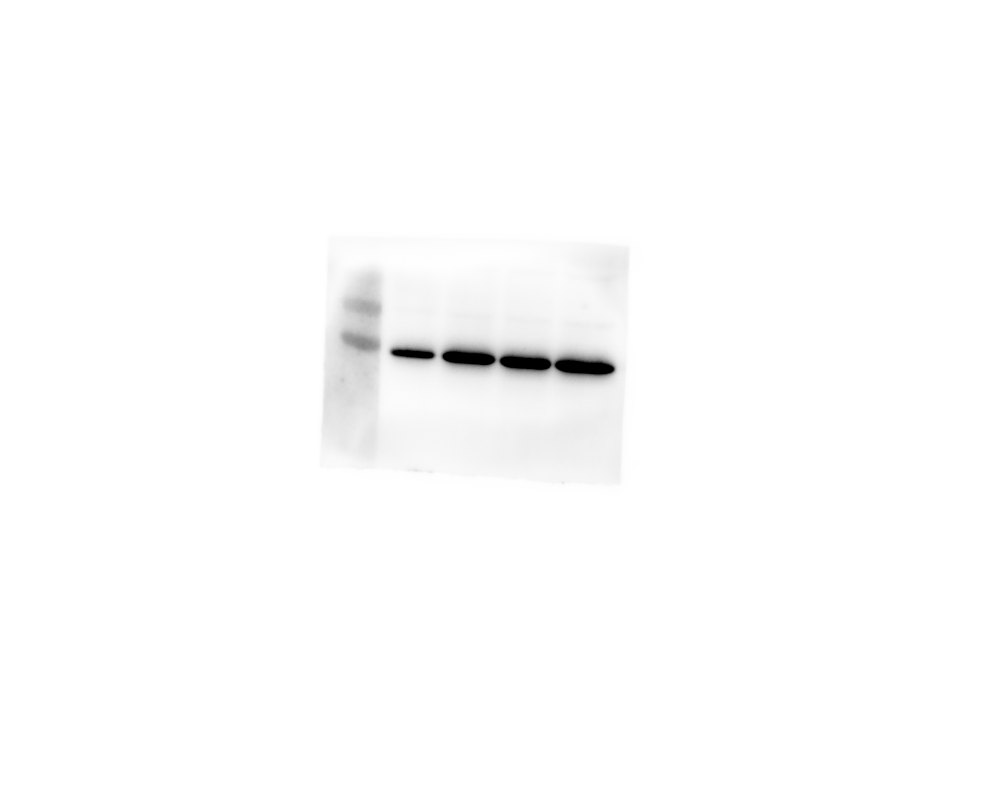


c-Myc


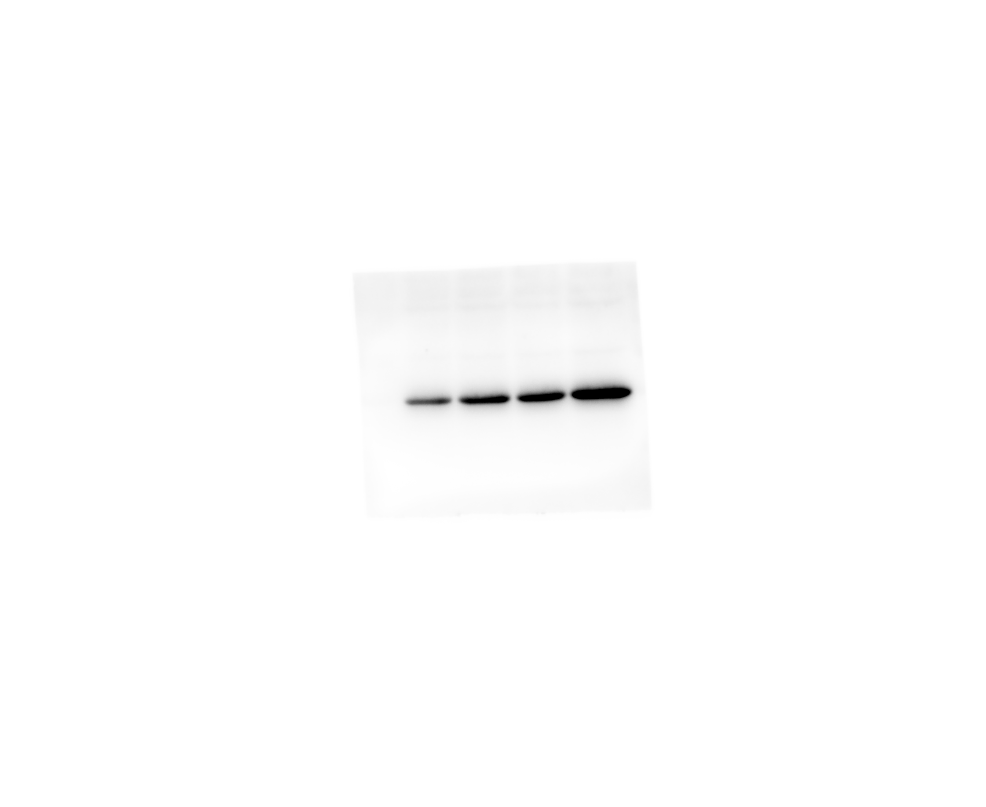


GAPDH


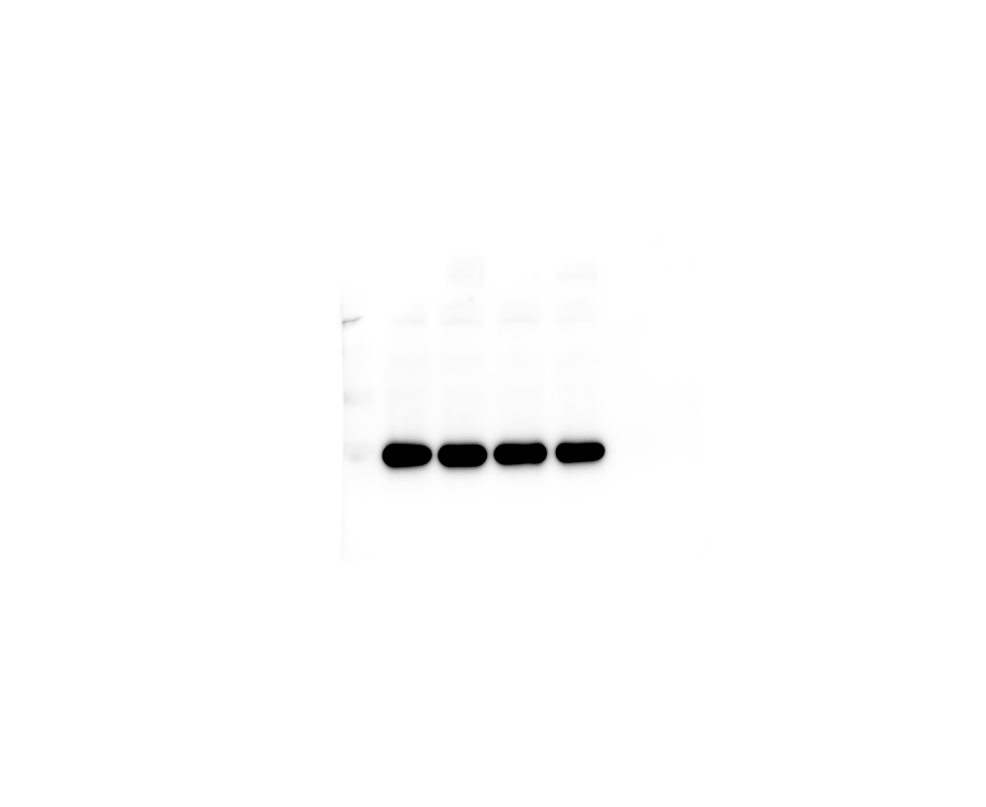


**Supplementary Figure 4**

Wnt1


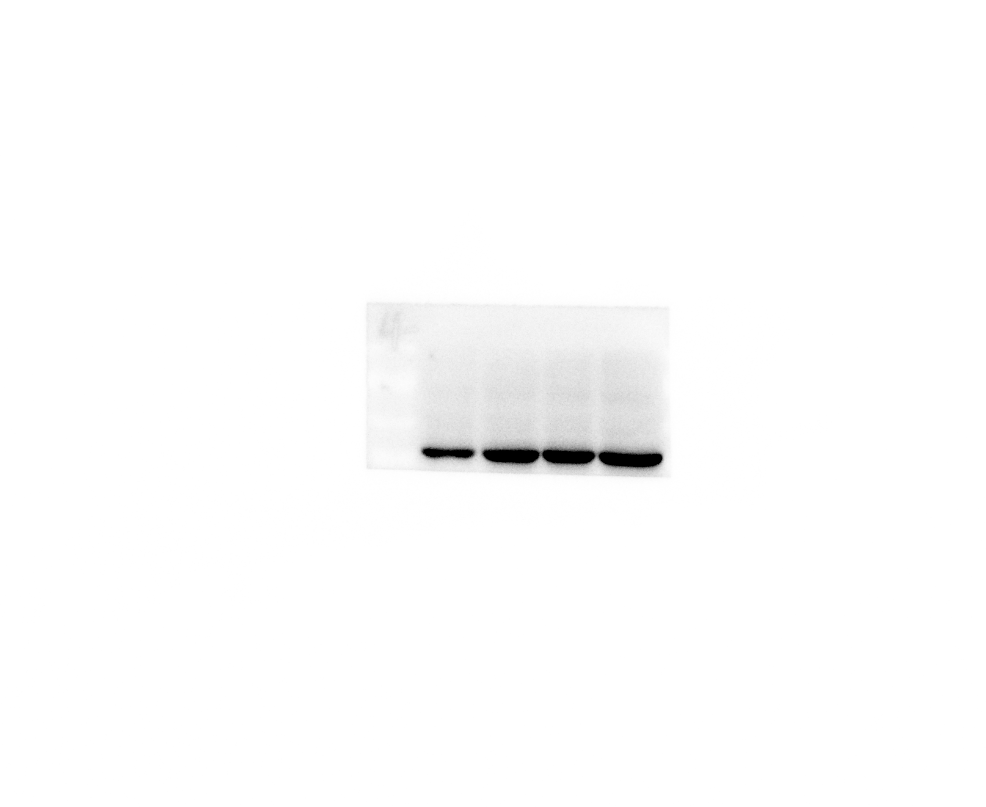


Wnt4


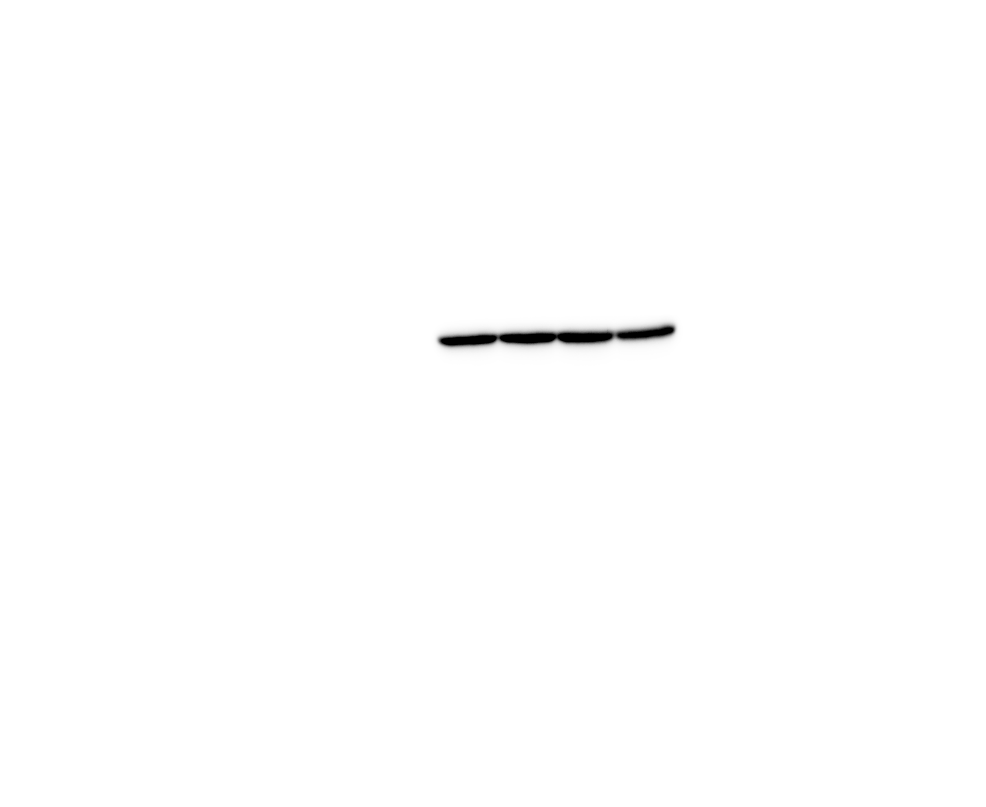


β-catenin


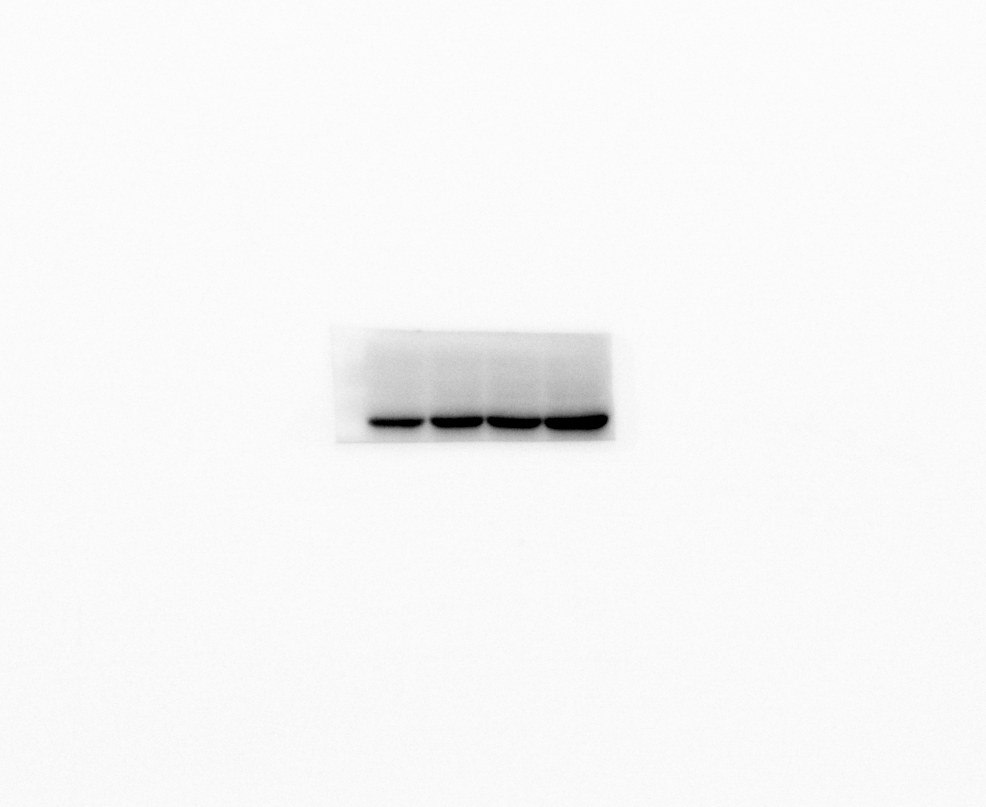


Cyclin D1


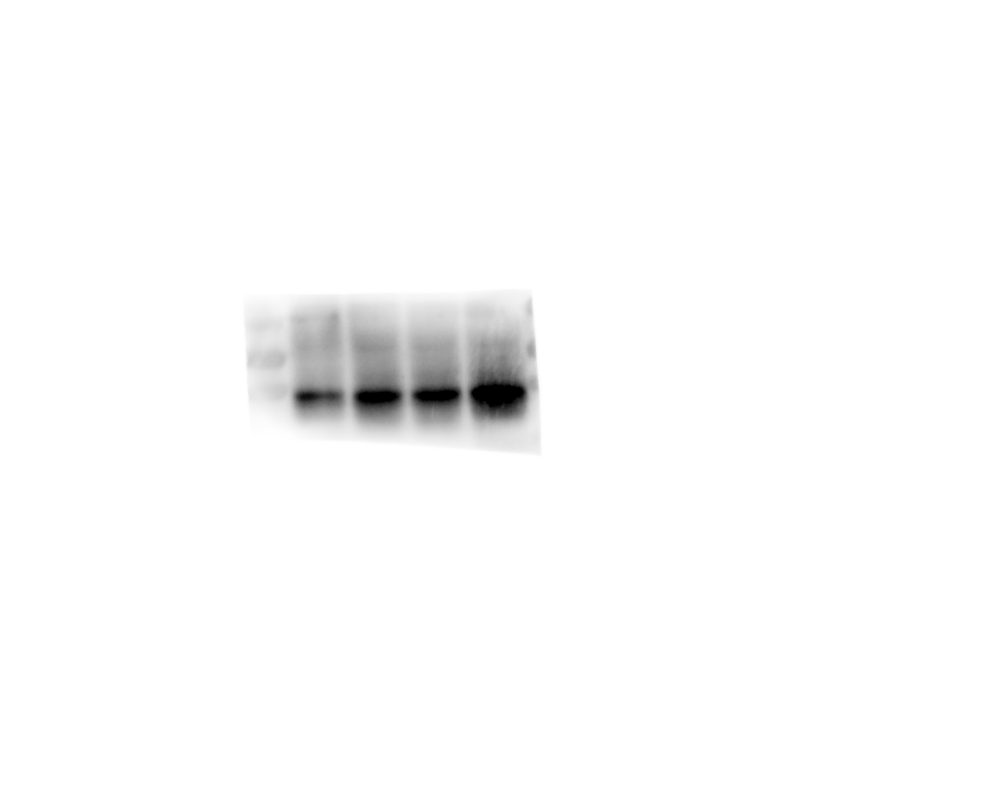


c-Myc


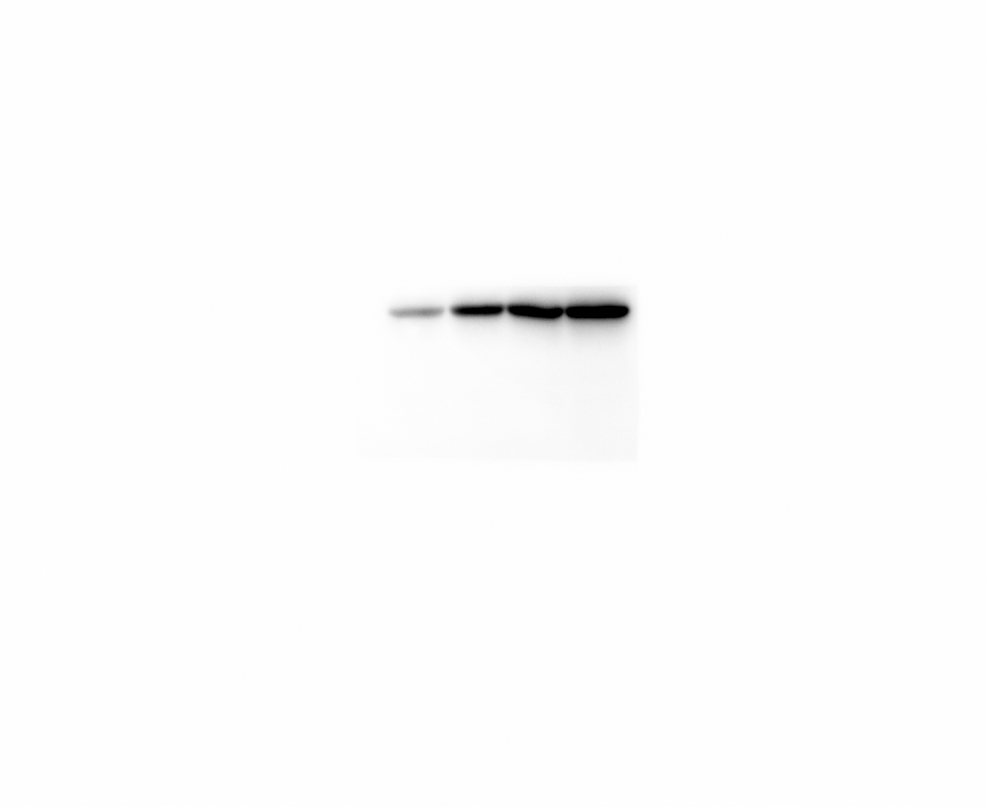


GAPDH


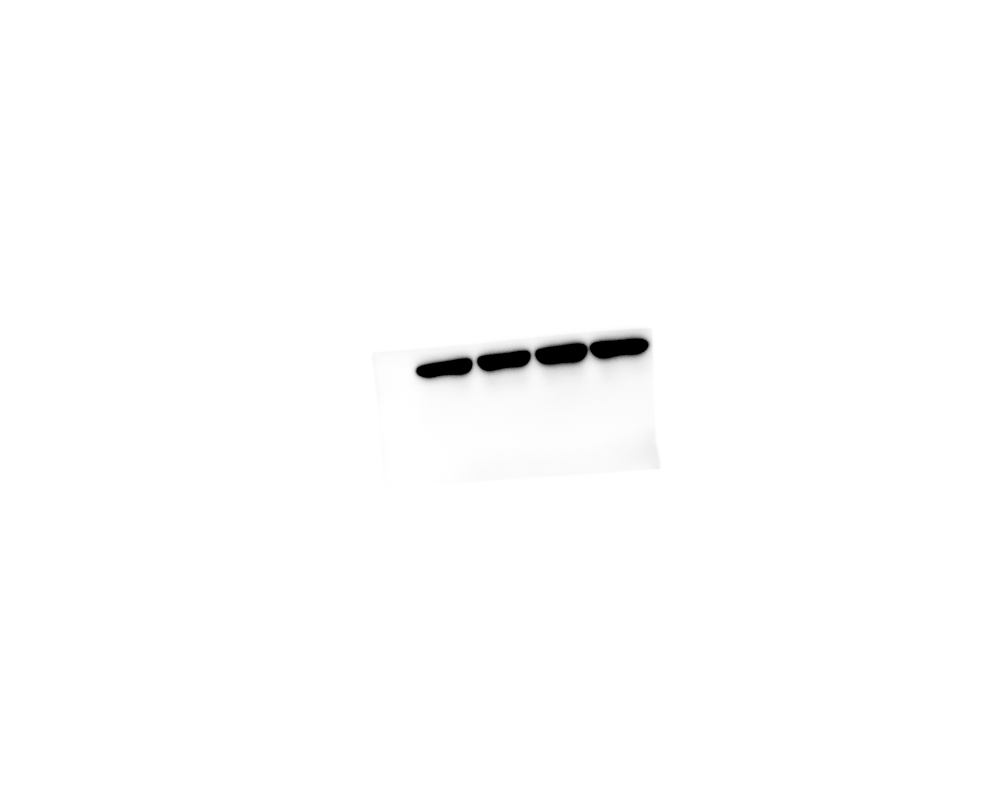

Supplement: Supplementary file 2 — Supplementary Material 2 [file 12885_2023_11639_MOESM2_ESM.docx]
